# Impact of Genetic Heterogeneity in Polymerase of Hepatitis B Virus on Dynamics of Viral Load and Hepatitis B Progression

**DOI:** 10.1371/journal.pone.0070169

**Published:** 2013-07-30

**Authors:** Chi-Jung Huang, Chih-Feng Wu, Chia-Ying Lan, Feng-Yu Sung, Chih-Lin Lin, Chun-Jen Liu, Hsin-Fu Liu, Ming-Whei Yu

**Affiliations:** 1 Graduate Institute of Epidemiology and Preventive Medicine, College of Public Health, National Taiwan University, Taipei, Taiwan; 2 Department of Gastroenterology, Ren-Ai Branch, Taipei City Hospital, Taipei, Taiwan; 3 Division of Gastroenterology, Department of Internal Medicine, National Taiwan University Hospital and National Taiwan University College of Medicine, Taipei, Taiwan; 4 Department of Medical Research, Mackay Memorial Hospital, Taipei, Taiwan; Yonsei University College of Medicine, Republic of Korea

## Abstract

**Objective:**

The hepatitis B virus (HBV)-polymerase region overlaps pre-S/S genes with high epitope density and plays an essential role in viral replication. We investigated whether genetic variation in the polymerase region determined long-term dynamics of viral load and the risk of hepatitis B progression in a population-based cohort study.

**Methods:**

We sequenced the HBV-polymerase region using baseline plasma from treatment-naïve individuals with HBV-DNA levels≥1000 copies/mL in a longitudinal viral-load study of participants with chronic HBV infection followed-up for 17 years, and obtained sequences from 575 participants (80% with HBV genotype Ba and 17% with Ce).

**Results:**

Patterns of viral sequence diversity across phases (i.e., immune-tolerant, immune-clearance, non/low replicative, and hepatitis B e antigen (HBeAg)-negative hepatitis phases) of HBV-infection, which were associated with viral and clinical features at baseline and during follow-up, were similar between HBV genotypes, despite greater diversity for genotype Ce vs. Ba. Irrespective of genotypes, however, HBeAg-negative participants had 1.5-to-2-fold higher levels of sequence diversity than HBeAg-positive participants (*P*<0.0001). Furthermore, levels of viral genetic divergence from the population consensus sequence, estimated by numbers of nucleotide substitutions, were inversely associated with long-term viral load even in HBeAg-negative participants. A mixed model developed through analysis of the entire HBV-polymerase region identified 153 viral load-associated single nucleotide polymorphisms in overall and 136 in HBeAg-negative participants, with distinct profiles between HBV genotypes. These polymorphisms were most evident at sites within or flanking T-cell epitopes. Seven polymorphisms revealed associations with both enhanced viral load and a more than 4-fold increased risk of hepatocellular carcinoma and/or liver cirrhosis.

**Conclusions:**

The data highlight a role of viral genetic divergence in the natural course of HBV-infection. Interindividual differences in the long-term dynamics of viral load is not only associated with accumulation of mutations in HBV-polymerase region, but differences in specific viral polymorphisms which differ between genotypes.

## Introduction

The natural history of chronic hepatitis B virus (HBV) infection has been divided into four phases: immune-tolerant (IT), immune-clearance (IC), non/low-replicative (LR), and hepatitis B e antigen (HBeAg) negative hepatitis (ENH) phases. The durations of these phases are variable among individuals with chronic HBV infection, and a spectrum of clinical severity has been observed [1].

HBV replicates by *reverse transcription using* an error-prone *polymerase lacking* proofreading ability [2], [3]. This error prone replication strategy leads to all possible point mutations within the viral genome during chronic infection [2]–[4]. The emergence of mutations within or flanking viral epitopes can impair T cell recognition or alter antigen processing, and consequently affects viral fitness and replication activity. Viral mutants with higher fitness levels may predominate by competitive replication, thus influence clinical consequence.

The natural course of HBV-infection depends on viral genetic divergence, the host defense strategies, and their interplay [5]. Evaluation of the spectrum of HBV genetic diversity is thus a necessary first step towards understanding the natural history of infection and interindividual heterogeneity in disease progression. In hepatitis C virus and human immunodeficiency virus, the accumulation of mutations due to host immune pressure and immune-driven escape mutations have been demonstrated to play important role in viral replication capacity, which facilitates studies of evolutionary forces and host-virus interactions involved in the pathogenesis of chronic viral disease [6]–[10]. So far, the understanding of viral sequence divergence that occurs during the dynamic course of chronic HBV infection and its impact on pathogenesis remains primitive.

In a 17-year longitudinal viral-load study, which was designed to describe the natural course of chronic HBV infection, we aimed to evaluate (i) the change of HBV sequence diversity in consecutive phases of natural history at the population level, (ii) the difference in the changing patterns of HBV genetic variation across phases of HBV-infection between HBV genotypes, (iii) the level of viral sequence divergence and viral polymorphisms in association with the long-term dynamics of viral load, as well as (iv) the risk for progression to hepatocellular carcinoma (HCC) and/or liver cirrhosis. We focused on a 2403-bp region encoding polymerase, which occupies 75% of the HBV genome. This region overlaps completely with the genes of three surface proteins, contains many T-cell epitopes, and plays an essential role in viral replication [2], [11]. Our results highlight changing virus-host interactions during the natural course of chronic HBV infection, and identify viral polymorphisms (or mutations), alone or in combination, that may be responsible for the dynamic nature of viral load and clinical outcomes.

## Methods

### The Cohort and Study Design

Study subjects were hepatitis B surface antigen (HBsAg)-positive and antibodies to hepatitis C virus-negative, who were selected from a previous longitudinal study on the long-term dynamics of plasma HBV-DNA levels and HCC, which included all incident cases of HCC ascertained by 2005 and a random sample of a subcohort that were chosen according to a case-cohort sampling design (n = 1143). The cohort was established in 1989–1992, when 2903 asymptomatic HBsAg-positive men aged 30–65 years who did not have HCC were enrolled during routine free physical examination at the Government Employee Central Clinics in Taipei, Taiwan [12], [13]. At enrollment, study participants completed questionnaires regarding lifestyle habits and medical history, and provided a blood sample. Participants were invited to attend annual follow-up examination with ultrasound and liver biochemical tests. Those who had abnormal liver biochemical tests or ultrasonographic features during follow-up were informed in a report of results and advised to receive further clinical evaluation or treatment. They returned for follow-up on a voluntary basis. The vital status and cancer development of participants were also investigated through linkage of the data of the National Death and Cancer Registry systems. In Taiwan, reimbursement under the National Health Insurance program for antiviral therapy for hepatitis B patients meeting certain criteria began on October 2003. Beginning in January 1, 2002, we expanded our follow-up interview questionnaire to include questions about any antiviral therapy the participant might have received. In performing case-cohort sampling from the full cohort, we excluded all persons who reported a history of antiviral therapy.

HBV-DNA quantification assay has been done on 7706 plasma samples collected during 16 years of follow-up from the 1143 subjects. The present study extended the follow-up period to December 31, 2006. Written informed consent was obtained from participants, and the research ethics committee at the College of Public Health, National Taiwan University approved this study.

Among the 1143 subjects, 1112 had data regarding the phase of HBV-infection. Sequencing was performed on 867 subjects with plasma HBV-DNA levels≥1000 copies/mL. After excluding samples that failed to produce a polymerase chain reaction (PCR) product or failed in sequencing, complete nucleotide sequence of the polymerase gene was available for 575 subjects (Table 1).

**Table 1:** Baseline characteristics and follow-up in study population^a^.

		Natural history of chronic HBV infection^b^			
	Total	IT	IC	LR	ENH
**Entire sample, n**	1143	86	23	932	71
Age, mean ± SD, yrs^c^	45.6±9.2	41.0±7.6	39.7±7.0	46.0±9.1	46.6±10.0
HBeAg positivity, n (%)	109 (9.8)	86 (100)	23 (100)	0 (0)	0 (0)
ALT, mean ± SD, U/L^c^	22.5±19.8	22.4±7.9	74.0±57.6	18.0±7.8	66.3±35.7
Log HBV DNA, mean ± SD, copies/mL^c^	4.54±1.97	8.33±1.75	8.36±1.27	4.07±1.49	4.86±1.78
HBV genotype^c^					
Genotype B, n (%)	872 (77.4)	51 (59.3)	9 (39.1)	744 (81.3)	44 (62.0)
Genotype C, n (%)	203 (18.0)	31 (36.1)	14 (60.9)	133 (14.5)	22 (31.0)
Genotype B+C, n (%)	51 (4.5)	4 (4.7)	0 (0)	38 (4.2)	5 (7.0)
BCP double mutations, n (%)^c^	370 (33.6)	22 (25.6)	10 (43.5)	296 (33.0)	31 (47.0)
Liver cirrhosis detected by ultrasonography, n (%)^c^	125 (11.6)	19 (23.2)	7 (31.8)	79 (9.0)	17 (27.0)
HCC, n (%)^c^	125 (10.9)	11 (12.8)	9 (39.1)	80 (8.6)	22 (31.0)
**Inclusion for sequence analysis**					
Plasma HBV DNA≥1000 copies/mL, n	867	83	23	674	59
Successful sequencing, n	575	64	13	443	36
Age, mean ± SD, yrs^c^	46.8±9.6	40.6±7.4	40.9±8.4	47.3±9.4	50.4±9.5
HBeAg positivity, n (%)	77 (13.8)	64 (100)	13 (100)	0 (0)	0 (0)
ALT, mean ± SD, U/L^c^	23.5±22.4	21.8±8.0	73.0±65.4	18.3±7.9	73.1±44.0
Log HBV DNA, copies/mL^c^	5.44±1.88	8.83±1.12	8.57±1.36	4.85±1.34	5.59±1.55
HBV subgenotype Ba/B3/Ce/Cs/D, nc,d	460/1/95/7/1	41/1/21/1/0	6/0/6/1/0	370/0/58/5/1	26/0/9/0/0
BCP double mutations, n (%)^c^	177 (31.1)	10 (15.6)	4 (30.8)	141 (32.0)	16 (48.5)
Liver cirrhosis detected by ultrasonography, n (%)^c^	75 (13.9)	11 (18.3)	5 (41.7)	47 (11.2)	10 (32.3)
HCC, n (%)^c^	68 (11.8)	4 (6.3)	5 (38.5)	44 (9.9)	14 (38.9)

ALT, alanine aminotransferase; BCP, basal core promoter; ENH, HBeAg negative hepatitis; HBeAg, hepatitis B e antigen; HBV, hepatitis B virus; HCC, hepatocellular carcinoma; IT, immune-tolerant; IC, immune-clearance; LR, non/low-replicative.

a Total number of subjects may vary because of missing value due to PCR/sequencing failure or nonparticipation in follow-up examination.

b 31 subjects with missing data on baseline ALT levels or HBeAg status were excluded from analysis of natural history.

c *P*<0.05 between the four phases of natural history determined with Kruskall-Wallis test (continuous variable) or χ^2^ test (categorical variable).

d 11 subjects had unclassified subgenotypes.

### Laboratory Analysis

Plasma HBV DNA and the basal core promoter (BCP) double mutations were assayed by PCR-based methods as described previously [12], [13]. HBV genotype was determined by multiplex PCR using the method described earlier [12] and reconfirmed by phylogenetic analysis. We performed nested PCR to amplify the polymerase gene from peripheral blood (for details, see Methods S1; primer design and amplification conditions are provided in Table S1).

The HBV sequences detected in this study that were included in the analysis of genetic divergence have been deposited in GenBank and assigned accession numbers KC792648 – KC793202.

### Sequence Analysis

One hundred and eighty-four reference sequences available on GenBank (http://www.ncbi.nlm.nih.gov/genbank/) were retrieved to obtain information on eight HBV genotypes (A-H) and 12 subgenotypes for use in sequence alignment and phylogenetic analysis. Maximum likelihood method was used with MEGA5 (http://www.megasoftware.net/) to evaluate the adequacy of assumptions made in models of nucleotide substitution (results from evaluating 24 major substitution models are provided in Methods S1 and Table S2). Maximum-likelihood phylogenetic tree was estimated using the best-fit model (GTR+I+G). We also inferred phyogenetic trees with the neighbor-joining method using the maximum composite likelihood estimate of the pattern of nucleotide substitution, which infers model-averaging phylogenies, and the Kimura 2-parameter model with the shape parameter of the γ distribution (K2+G). The complementary use of the additional method will help to understand the robustness of subgenotype classification under different model assumptions. Bootstrap analysis of 1000 replicates was applied to assess the reliability of individual nodes for each phylogenetic tree.

Three parameters of genetic divergence were calculated under proper nucleotide substitution models available on MEGA5: genetic distance, the number of synonymous substitutions per synonymous site (dS), and the number of nonsynonymous substitutions per nonsynonymous site (dN). In addition to the entire polymerase gene, we examined whether overlapping and nonoverlapping reading frames differ in genetic diversity across phases of HBV-infection. Genetic distance was calculated under *the* K2+G model, while dS and dN were estimated by using the K2 correction model (Kumar’s method). Stratified analyses according to predominant subgenotypes (Ba or Ce) were performed to account for phylogenetic relationships. All parameters of genetic divergence for each sequence were calculated via pair-wise comparison with the population consensus sequence (for details about the estimation of consensus sequence, see Methods S1). The Shannon entropy of a nucleotide position was calculated for determining mutant spectrum complexity using BioEdit v7.1.3.0 (http://www.mbio.ncsu.edu/bioedit/bioedit.html).

### Statistical Analysis

We assessed the influences of viral genetic divergence and single nucleotide polymorphisms (SNPs) on the levels of viral load, both cross-sectionally and longitudinally. In the cross-sectional analysis, we related factors with baseline viral load using multivariable linear regression, and partial *R^2^* were calculated. In longitudinal analysis, we used linear mixed model to analyze factors associated with change in viral load over time. Multiple logistic regression was used to evaluate associations between viral SNPs and clinical outcomes. For a subset of viral SNPs discovered to be associated with viral load with *P*≤0.01, principal component (PC) analysis was applied to evaluate multivariate SNP correlations to infer clusters of viral load-associated SNPs.

## Results

### Natural History, Dynamics of Viral Load, and Disease Progression

**Figure 1:**
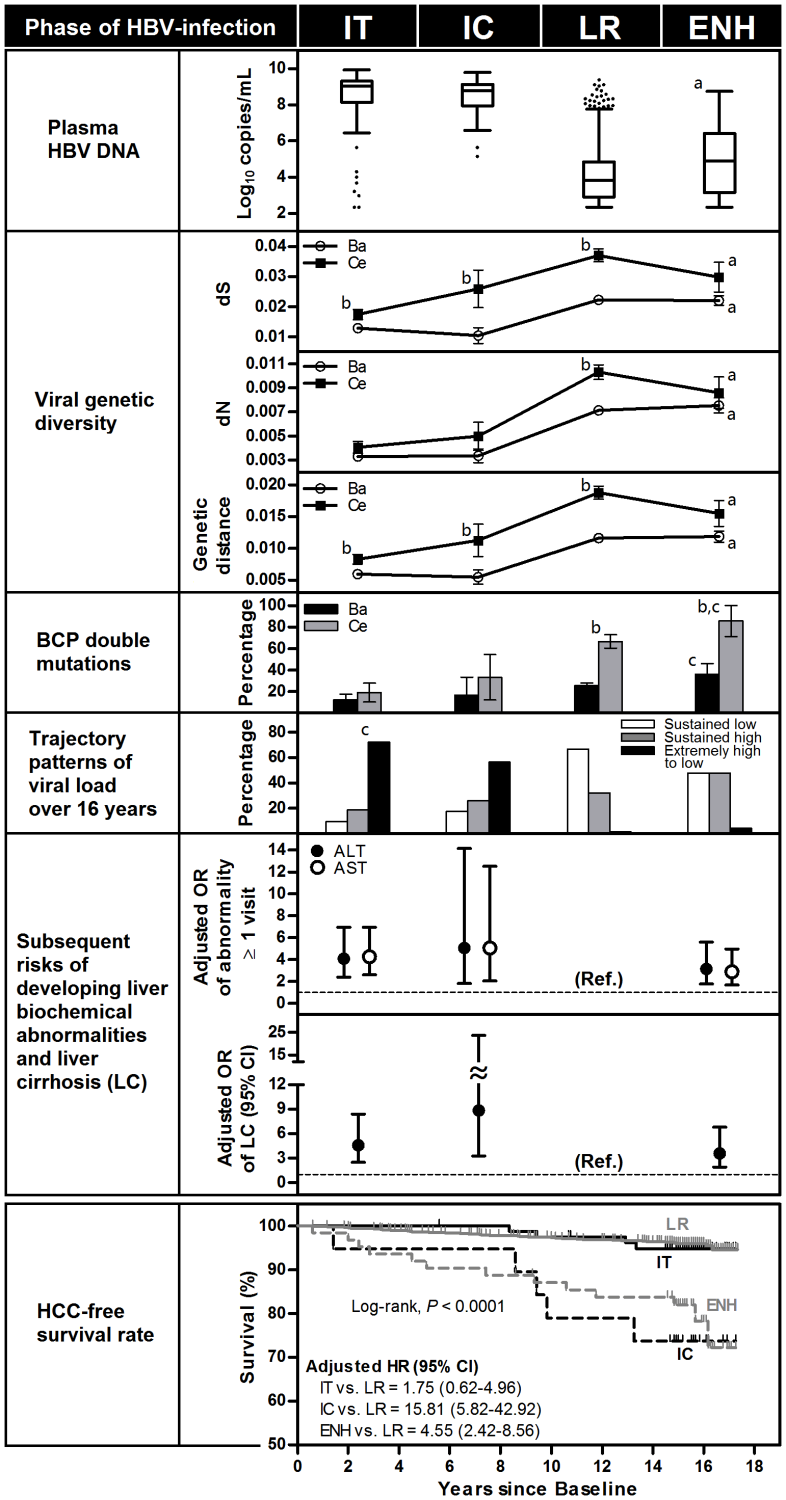
Viral and clinical features in association with the four phases of chronic HBV infection. The analysis was performed using data from a case-cohort study of participants with chronic HBV infection aged 30–65 y at recruitment in 1989–1992 (n = 1112), and followed to 2006. Parameters of viral genetic diversity were measured in 460 subjects with HBV/Ba and 95 with HBV/Ce (GenBank accession numbers KC792648 – KC793202). Data on BCP double mutations were available for 441 subjects with HBV/Ba and 91 with HBV/Ce. There are three trajectory classes for the time trend of viral load: “sustained low”, “steadily high” (consistently in the levels of 5–6 log_10_ copies/mL), and “extremely high to low” (gradually declining from the levels of 8–9 log_10_ copies/mL), as defined by our previous longitudinal viral-load study [13]. Disease-free survival rate (i.e. cumulative incidence) and hazard ratio (HR) for hepatocellular carcinoma (HCC) were estimated by the Kaplan-Meier method and Cox regression model, respectively, using the subcohort of 1054 participants. Odds ratios (ORs) and HRs were derived by multivariate models adjusted for age, cigarette smoking, alcohol consumption, and body mass index. ^a^ *P*<0.0001 for IT/IC vs. LR/ENH. ^b^ *P*<0.0340 for Ba vs. Ce subgenotype. ^c^ *P_trend_*<0.0210 across phase of HBV-infection, determined by Mantel–Haenszel extension of the χ^2^ test for trend.

Based on recommended criteria of the phases of HBV-infection [14], [15], serological profiles of HBeAg serostatus, serum alanine aminotransferase (ALT) levels, and measurement of HBV DNA were used to divide subjects into a phase of HBV-infection: IT (HBeAg positive, normal ALT, high viral load), IC (HBeAg positive, ALT>upper limit of normal [ULN], high viral load), LR (HBeAg negative, normal ALT, low or intermediate levels of viral load), and ENH (HBeAg negative, ALT>ULN, intermediate levels/often fluctuating viral load). HBeAg serostatus and serum ALT levels were used as essential criteria to define the phases of HBV-infection, and viral load was used as secondary criterion. Subjects in the IT/IC phase were younger and more likely to be infected with genotype C HBV, and had lower prevalence of BCP double mutations than those in the LR/ENH phase (Table 1 and Figure 1, other characteristics are shown in Table S3).

The percentages of liver biochemical abnormalities at a follow-up visit among the IT/IC subjects were significantly higher than the LR subjects and only slightly and nonsignificantly higher than the ENH subjects (Table S3); however, IC and ENH phases were associated with more advanced liver diseases. The 1112 subjects comprise a subcohort of 1054 subjects, which contained 65 HCC incident cases. Using the subcohort we estimated that the hazard ratios were 1.75 (95% CI: 0.62–4.96), 15.81 (95% CI: 5.82–42.92), and 4.55 (95% CI: 2.42–8.56) for IT, IC, and ENH, respectively, compared to LR as the reference group (for 15-year cumulative incidences, see Results S1), after adjusting for multiple putative risk factors of HCC. There was also a highly significant association between the phase of HBV-infection and liver cirrhosis detected by ultrasonography during follow-up. The odds ratios, based on the 1112 subjects, were estimated as 4.61 (95% CI: 2.52–8.43), 8.85 (95% CI: 3.31–23.68), and 3.62 (95% CI: 1.92–6.82), respectively, for IT, IC, and ENH compared with LR (Figure 1). The results are similar for the subjects with sequence data.

### Changes in Viral Genetic Diversity and the Occurrence of BCP Double Mutations in Association with Phase of HBV-infection for Subgenotypes Ba and Ce

Classification of subgenotypes based on different nucleotide substitution models differs by only one sequence which revealed the mixed genotype infections of genotype B and C in our previous multiplex PCR [12]. Considering model uncertainty and the potential limitation of direct sequencing in accurate determination of mixed genotype, the subgenotype of this subject was justified as unclassified subgenotype. Among the 575 subjects with sequence data, 460 (80.0%) were Ba, 95 (16.5%) were Ce, 7 (1.2%) were Cs, and 13 (2.3%) had other or unclassified subgenotypes (Table 1).

As shown in Figure 1, regardless of subgenotype, dS is 3-to-5-fold higher than dN across phase of natural history. The more striking dS/dN ratio occurred in the two nonoverlapping subregions of the polymerase gene (Figure S1), suggesting negative selection for these regions of this gene. In HBV/Ba, the prevalence of the BCP double mutations increased from 12.2% in IT to 16.7% in IC, and then further increased to 25.8% in LR and 36.0% in ENH (*P_trend_* = 0.0203); in HBV/Ce, the prevalence of the BCP double mutations increased from 19.1% in IT to 33.3% in IC, and then further increased to 66.7% in LR and 85.7% in ENH (*P_trend_*<0.0001). Numbers of nucleotide substitutions, genetic distance, and the prevalence of BCP double mutations in each of the four phases of HBV-infection were consistently lower for HBV/Ba than for HBV/Ce (all *P*<0.02 for comparisons between subgenotypes in the analysis unstratified by phases of HBV-infection). Nevertheless, both subgenotype groups revealed quite similar patterns of changes in the estimates of viral divergence across phases of HBV-infection. Strikingly, all the estimates of viral divergence away from population consensus sequence for the sequence region showed a dramatic 1.5-to-2 fold rise in moving from IT/IC to LR and then stabilized (Figure 1). Also, except for the short fragment within the 250-bp overlapping region of polymerase and X, we found a similar changing pattern of genetic divergence across phases of HBV-infection between the largest overlapping reading frame and two nonoverlapping reading frames, despite of regional difference in genetic diversity (Figure S1).

### Levels of Viral Sequence Divergence from the Consensus Sequence and Dynamics of Viral Load in Subgenotypes Ba and Ce

**Figure 2:**
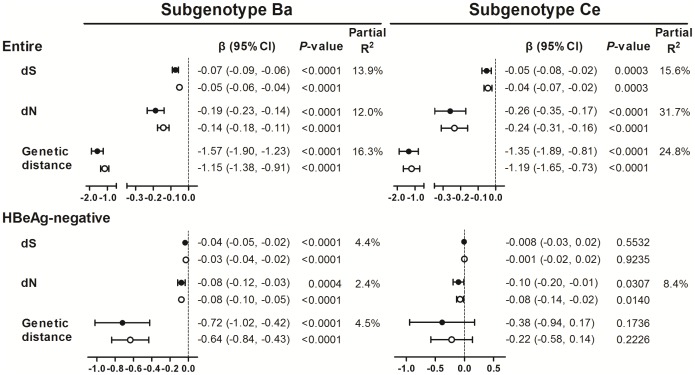
Influence of viral genetic diversity on hepatitis B viral load by HBV subgenotypes. The plot show the estimated impact (β estimates; regression coefficients) per 1-unit increment of dS (10^−3^ substitution per site), dN (10^−3^ substitution per site), or genetic distance (10^−2^ nucleotide substitution) on cross-sectional (solid circle and horizontal line) and longitudinal (empty circle and horizontal line) measures of viral load (log copies/mL) and 95% confidence intervals (CIs). All regression models include age as a covariate. The partial *R^2^* values measure the marginal contribution of each parameter of viral genetic diversity to the variability in baseline viral load when age was already in the respective linear regression model.

Viral sequence diversity is shaped by a combination of mutation and immune-mediated selection forces. We thus further examined the correlation between levels of viral divergence away from the population consensus sequence and viral load. All estimates of viral divergence were statistically, inversely associated with cross-sectional and longitudinal measures of viral load after adjustment for age in both genotype groups (*P*<0.0004). These estimates account for 12%–32% of the baseline viral load variability, on top of partial *R^2^* explained by dN in the group with HBV/Ce. Approximately 90% of the study subjects were HBeAg negative at baseline; most of whom were in the phase of immune control with lower levels of viral load. Contrary to the first analysis, the effect size estimates in terms of regression coefficients and the partial *R^2^* values of the respective linear regression models appear smaller among HBeAg-negative subjects, although estimates of genetic divergence, especially dN, remained statistically significantly associated with viral load in each genotype group (Figure 2).

### Divergent Profiles of Viral SNPs in Association with the Dynamics of Viral Load between Subgenotypes Ba and Ce

**Figure 3:**
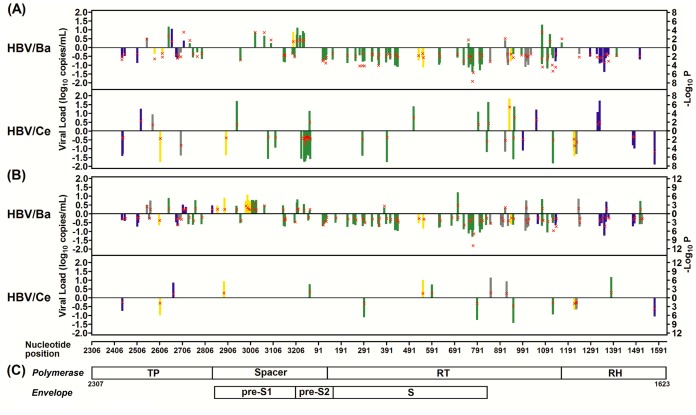
Map of viral SNPs associated with long-term hepatitis B viral load by HBV subgenotypes. Shown are the estimated impacts of viral SNPs on longitudinal viral load (regression coefficients; bar) and their corresponding *P* values (red × symbol) in the entire subjects (A) and in the HBeAg-negative subjects (B) with sequence data, as well as the structure of the genes across a 2403-bp stretch in the sequence region covering HBV polymerase, pre-S1, pre-S2, and surface (S) (C). Viral SNPs are marked as colored bars if their locations fall in a region within or flanking (defined as occurring within 3 aa apart from the epitope) known HLA class I (blue)- or class II (yellow)-restricted epitopes (class I plus II, green) (source: http://www.immuneepitope.org/); otherwise viral SNPs are shown as gray bars.

All the polymorphic nucleotide sites at which the number of subjects that carried the variant type was >5 along the 2403-bp stretch of sequence region were tested for association with longitudinal viral load. We identified 153 viral SNPs in total for both subgenotypes with *P*<0.05. Only seven viral SNPs were common between subgenotypes Ba and Ce. In HBV/Ba, we found 88 SNPs showing negative associations and 19 showing positive associations. In HBV/Ce, we found 41 SNPs showing negative associations and 12 showing positive associations. Notably, approximately 90% (95 of 107 for HBV/Ba and 49 of 53 for HBV/Ce) of these identified SNPs fell in a region within or flanking previously defined T-cell epitopes (Figure 3A and Table S4). We also evaluated the impacts of viral SNPs on viral load in HBeAg-negative participants who had a wide range of viral load, presumably reflecting different virus-host interactions, and found viral SNP profiles that differed from the profiles identified in the entire samples with sequence data (Figure 3B and Table S4).

There may be covarying nucleotide positions that involve the maintenance of biologically relevant structures and functions and perhaps reflect coevolution. We next evaluated the cluster structure of a subset of viral load-associated SNPs with *P*≤0.01 by principal component analysis. In many instances, clustered viral SNPs were associated with amino acid (aa) substitutions within or flanking the same epitope and the phase of HBV-infection (Figure S2 and Table S5).

### Identification of Virulence Markers for Disease Progression

Since HBV strains that occur after strong immune-driven natural selection during HBeAg seroconversion may carry escape mutations that can adapt in the host, we sought to identify virulence markers which enhance viral replication and disease progression in HBeAg-negative subjects. In addition to the BCP double mutations, seven viral load-associated viral SNPs in the polymerase region identified in HBeAg-negative subjects had significant associations with increased viral load (Figure 3B and Table S4) and risks for incident HCC and/or liver cirrhosis (Table 2). All but one of these seven viral SNPs were identified in the group with HBV/Ba, in which these SNPs had frequencies 0.7%–2.8% in unaffected subjects and 9.1%–15.2% in the incident cases of HCC. The association between each of these SNPs and increased risk for HCC remained significant in HBV/Ba subjects after adjusting for the BCP double mutations and other putative risk factors for HCC. The ORs of carrying any of the six viral SNPs were 10.12 (95% CI = 4.24–24.16) for HCC and 3.58 (95% CI = 1.58–8.09) for liver cirrhosis.

**Table 2:** Viral polymorphisms associated with increased viral load in HBeAg negative phase and progression to HCC and/or liver cirrhosis (LC) by HBV subgenotypes.

			Impact on viral load		% Variant types				% Variant types			
Position	Consensusnt type	Variantnt type^a^	β (change in log HBV DNA copies/mL)^b^	P-value	HCC	Non-HCC	OR^c^	(95% CI)	LC	Non-LC^d^	OR	95% CI
Subgenotype Ba, n					33	427			44	390		
Nt3031 (pol-spacer/pre-S1)	G	A/R/K/del	0.78	0.0494	9.1	0.9	9.81	(1.58–60.95)	4.6	1.0	5.17	(0.76–35.36)
Nt3150 (pol-spacer/pre-S1)	A	C/G/T/R/del	0.47	0.0432	15.2	2.8	5.43	(1.53–19.23)	11.4	2.8	4.48	(1.33–15.09)
Nt3211 (pol-spacer/pre-S2)	T	A/Y/W/del	0.65	0.0257	12.1	1.6	4.29	(1.04–17.70)	9.1	1.5	4.06	(0.99–16.63)
Nt3213 (pol-spacer/pre-S2)	G	A/del	0.82	0.0419	12.1	0.7	7.52	(1.46–38.78)	9.1	0.5	11.89	(1.86–75.97)
Nt27 (pol-spacer/pre-S2)	A	R/M/W/del	0.55	0.0588	15.2	2.1	4.81	(1.34–17.33)	13.6	1.8	5.10	(1.47–17.71)
Nt1008 (pol-RT)	T	C/G/Y/K/D	0.76	0.0102	9.1	2.1	5.49	(1.22–24.74)	2.3	2.6	0.86	(0.10–7.58)
Cumulative association												
No. of pol/pre-S SNPs			0.34	0.0002			2.26	(1.34–3.81)			2.25	(1.31–3.87)
(per one increment)												
No. of pol/pre-S SNPs and BCPdouble mutations												
No. of pol/pre-S SNPs	0		1.00	(referent)	54.6	92.3	1.00	(referent)	72.7	91.3	1.00	(referent)
	1		0.62	0.0001	33.3	6.6	10.12	(4.24–24.16)	18.2	7.7	3.58	(1.58–8.09)
	≥2		0.67	0.0502	12.1	1.2			9.1	1.0		
BCP double mutations	Wild	Variant type	0.35	0.0007	46.9	23.7	2.52	(1.06–6.00)	55.8	22.4	3.91	(1.97–7.76)
Subgenotype Ce, n					30	65			26	62		
Nt552 (pol-RT/S)	T	C/Y	1.00	0.0291	23.3	0.0^e^	NA		19.2	1.6	10.68	(1.06–107.36)

HBeAg, hepatitis B e antigen; HBV, hepatitis B virus; HCC, hepatocellular carcinoma; NA, not applicable; Nt, nucleotide; Pol, polymerase; RT, reverse transcriptase; S, surface.

a R, A/G heteroduplex; K, T/G heteroduplex; Y, C/T heteroduplex; W, A/T heteroduplex; M, A/C heteroduplex; D, A/G/T heteroduplex. del: deletions involved in risk for disease progression range in size from 1 to 1049 nucleotides (Figure S3).

b Results were derived from a linear mixed model adjusting for age.

c Odds ratios (ORs) and 95% confidence intervals (CIs) were mutually adjusted for age, cigarette smoking, alcohol consumption, body mass index, and the BCP double mutations, except for ORs in the HBV subgenotype Ce group.

d 26 subjects with Ba subgenotype did not participate in follow-up ultrasonography examination.

e *P* = 0.0002 derived from Fisher’s exact test for comparison between HCC cases and non-HCC controls.

In each position of the seven viral SNPs, the Shannon entropy value was at least 3-fold higher in participants with progression to HCC than in non-progressors (Table S6). The HBV polymerase gene is a complex genomic region with overlapping functions. We then examined the effect of each of these polymorphisms on aa sequence. Six of the seven SNPs were found to alter the polymerase aa sequence. In addition, 5 are predicted to change the overlapping pre-S aa sequence, and 1 also leads to aa change in the overlapping S region (Table 3).

**Table 3:** Effects of viral SNPs in relation to enhanced viral load and HCC on coding function by HBV genomic regions.

Nt Site	Polymerase				Envelope (Pre-S/S)			
	aa site (consensus type)	Variant type		Functionaldomains	aa site^a^(consensus type)	Variant type		ORF/functional domains
		HCC	Non-HCC			HCC	Non-HCC	
**Subgenotype Ba**								
3031	242 (S)	N/I/del	N/G/del	Spacer	62 (A)	T/S/del	T/del	Pre-S1/transactivator domain
3150	282 (R)	G/W/**del** b	G/K/T/del	Spacer	101 (S)	T/**del**	T/A/L/del	Pre-S1/Hsc70, CAD & S-promoter
3211	302 (V)	**del**	A/E/L/M/del	Spacer	122 (W)	**del**	R/L/del	Pre-S2/nucleocapsid-binding site, viral secretion & pHSA
3213	303 (E)	**del**	A/D/G/K/del	Spacer	122 (W)	**del**	R/L/del	Pre-S2/nucleocapsid-binding site, viral secretion & pHSA
27	312 (S)	**A**/**del**	A/T/P/del	Spacer	132 (Q)	P/L/**del**	H/P/R/del	Pre-S2/pHSA
1008	639 (L)	Silent (L)	I	RT				
**Subgenotype Ce**								
552	487 (Y)	F	None^c^	RT	307 (M)	**T**/L/S	None	S/‘a’ determinant

aa, amino acid; CAD, cytosolic anchorage determinant; HBV, hepatitis B virus; HCC, hepatocellular carcinoma; Hsc70, heat shock protein 70-binding site; Nt, nucleotide; ORF, open reading frame; pHSA, polymerized human serum albumin-binding site; RT, reverse transcriptase; S, surface; SNPs, single nucleotide polymorphisms.

a Based on deduced aa site of the HBV large envelope protein.

b Specific types of SNPs (deletions or aa substitutions) exhibiting significantly altered frequency (determined by Fisher’s exact test) in HCC cases, as compared with non-HCC controls, are shown in bold.

c All shown as consensus aa type.

## Discussion

In a cohort of antiviral treatment-naïve individuals with chronic HBV infection followed for 17 years, we found a remarkable association for the phases of chronic HBV infection determined at baseline with trajectories over time for repeated measures of viral load and different biochemical and ultrasound liver abnormalities. The serological profiles with respect to HBeAg status, ALT levels, and HBV DNA change with transition through the different phases of HBV-infection. By using follow-up study, it has been reported that there is a positive association between ALT levels and the cumulative HBeAg seroconversion rate. Hepatitis B patients may often have repeated episodes of liver biochemical abnormality before eventually achieving HBeAg seroconversion [16]. According to our data, the majority (60–70%) of IT subjects experience transient ALT or AST elevation during long-term follow-up, which may be associated with a transition to the IC phase characterized by fluctuating or high HBV-DNA and ALT levels and increased inflammatory activity in the liver [1], [14]. The high risks for developing both HCC and liver cirrhosis in IC- and ENH-subjects compared with LR-subjects is compatible with clinical concepts related to the natural history of HBV-infection [1], [14]. Using the database, we were thus possible to address the impact of viral genetic heterogeneity on the dynamics of HBV viral load and hepatitis B progression during chronic infection.

We generated HBV sequence data for genotypes B and C that differ by ≥8% at the nucleotide level [2], and found greater viral genetic diversity across genes in the sequence region in HBV/Ce than in HBV/Ba subjects. Sequence divergence could influence immunogenic patterns, thereby resulting in divergent selective pressures and differences in evolutionary adaptability between the genotypes [7], [17]. Genotype B is associated with an earlier and more frequent HBeAg seroconversion and a shorter duration of sustained high viral load than genotype C [12], [18], suggesting more immunogenic and susceptible to host immunity for genotype B than for genotype C. Our finding may strengthen the diverse mechanisms by which viral evasion of immune responses may be effectively achieved for genotypes B and C, and the potential role of genotype sequence variation in these processes.

Regardless of genotypes, however, there was a striking increase in viral genetic diversity in the LR/ENH-subjects, when compared with the IT/IC-subjects. This phenomenon was observed in both overlapping and nonoverlapping reading frames, and is consistent with researches of viral quasi-species evolution in the BCP/precore or partial core region of HBV, which demonstrated that viral sequence diversity was increased to 1.5-to-2.4 fold after HBeAg seroconversion, a key event associated with progression to the immunoactive phase [19], [20]. The dramatic viral evolutionary shifts after HBeAg seroconversion underline the necessity to consider the role of viral genetic divergence in clinical outcomes. Importantly, we also found a significant, dose-dependent inverse association with viral load for the levels of viral genetic divergence apart from the population consensus sequence, which are estimated by the number of nucleotide substitutions and genetic distance. This association was found irrespective of HBV genotypes, suggesting that overwhelming immune selection may dominate effective control of HBV.

HBV viral load is highly variable, and many factors certainly contribute to the large unexplained portion of the interindividual variability. Our data indicate that the levels of viral genetic divergence explains 12–32% of the total observed variability in cross-sectional measures of baseline viral load in a large population. These fractions compare favorable to what is known for other factors, such as demographic and viral genotype, which often explain only a few percent of the variance [12], [13], [21]. However, more work is needed to understand extra predictive value of immunologic factors and complex interactions between virus and host.

By performing longitudinal analysis of repeated measures of viral load, we are able to confirm and extend the cross-sectional (baseline) findings to indicate that the level of viral genetic divergence was an independent predictor for the longitudinal viral load. The magnitude of the association between levels of sequence divergence and viral load in the longitudinal analysis was very close to that observed in the cross-sectional examination of baseline levels of viral load, but was slightly attenuated perhaps due to cumulative effects of intraindividual variability and error measurements of viral load.

The mechanisms by which greater numbers of nucleotide substitutions are associated with lower viral load over the next several years remain elusive. Since HBeAg seroconversion leads to increased genetic diversity of HBV, which is accompanied by remarkable decrement of viral load, the inverse association between levels of viral genetic divergence from the consensus sequence and longitudinal viral load would be attributed, at least partly, to the vigorous immune responses during the transition from IC to LR. However, we also observed an inverse association between either dN or dS and viral load in HBeAg-negative subjects, which are a heterogeneous group with different levels of viral load, clinical course and prognosis [1], [12]–[14], [18]. There might be, therefore, differences in the dynamics of HBV sequence variation in response to variable immune selective pressures after HBeAg seroconversion. Despite a paucity of data, it has been reported that the presence of effective immune response contributes to control virus replication among the large majority of HBeAg-negative individuals with chronic HBV infection lacking evidence of liver damage [22].

In pathogenesis of other chronic viral infections, some escape mutations that pose different fitness levels have been established as critical factors [6], [23]–[25]. However, studies of HBV mutations with relevance to evolution, viral replication activity, and disease pathogenesis have been limited [13], [26]–[28]. In this study, we initially sought clues to mutations that are likely to increase or decrease viral replication by screening viral SNPs. Second, we tested the hypothesis whether viral SNPs enhance viral replication activity, thus leading to the development of HCC and liver cirrhosis.

Here, we revealed for the first time a profile including 153 viral SNPs that were associated with longitudinal viral load from analysis of a large genomic region of HBV, with distinct signatures found for HBV genotypes B and C. Further extensive functional analysis should be required for understanding the mechanisms of action of these complex HBV variants on the viral replication activity. Regardless of whether there is evidence for the biological significance of identified viral SNPs, however, it is of another interest that the majority of these viral SNPs associated with viral load fall in areas within or flanking T-cell epitopes. We also examined viral load-associated SNPs for covarying sites, and show that clustered SNPs both within and proximal to epitopes correlate with the phase of HBV-infection. This suggests that there might be coordinated evolution of multiple nucleotide positions under selective pressure. Unlike most previous studies that simply demonstrate HLA binding affinity to define HBV epitopes by using synthetic peptides, which does not necessarily correlate with host response or clinical outcome [11], [29], our approach enables the large-scale screening of specific sequence variations in HBV epitopes that could correlate with clinical consequences. However, other researches will be needed to assess how these viral SNPs identified influence T-cell function.

The significant immune alterations during HBeAg seroconversion can lead to a wide range of sequence polymorphisms in relation to viral fitness and virulence. The observation that the majority of viral SNPs showed an inverse association with longitudinal viral load in HBeAg-negative subjects suggests that many escape mutations might occur at the expense of viral fitness. This might explain that the large numbers of viral SNPs seemed to be primarily associated with viral load and were not determinants of disease progression. Notably; however, our data indicate that BCP double mutations were gradually accumulated during chronic infection and associated with increased viral load. Our findings also support previous observations indicating a positive association between BCP mutations and worse clinical outcomes [13], [27], [28]. In addition, we identified seven viral SNPs in the polymerase region that were associated to both enhanced viral load and risks for HCC even after adjustment for multiple putative HCC risk factors and the BCP double mutations. All but one of these SNPs were found in the HBV/Ba group, which had lower prevalence of BCP double mutations as compared to HBV/Ce. Similar to the evolutionary behavioral phenotype of BCP mutations [28], these viral SNPs have a major impact on viral replication levels and risks of advanced liver diseases after HBeAg seroconversion.

Our prospective study design and long-term follow-up indicate that these viral SNPs are associated with the risk of developing HCC instead of just the presence of the malignancy. Despite of infrequently occurring in participants who did not progress to HCC, these viral SNPs were enriched in those who progressed to HCC. In HBV/Ba, carriage of any of the six identified viral SNPs confers a 10-fold increase in risk for HCC. Because many of these SNPs were also associated with liver cirrhosis, the associations between these SNPs and HCC are likely to be biologically significant.

In addition, all but one of these viral SNPs associated with HCC lead to aa substitutions/deletions at sites in the overlapping reading frames of polymerase and pre-S/S. Five of these SNPs locate in a pre-S region with frequent deletions observed in progressive liver diseases, and are associated with specific aa deletions or changes in multiple functional domains involved in RNA transcription, virus replication, or virion assembly and secretion [30]. The only genotype C-related SNP associated with HCC alters the aa sequence within the ‘a’ determinant of the S region, a major antigenic determinant [31]. Furthermore, in each position of the seven SNPs participants who progressed to HCC showed 3-fold or greater heterogeneity of nucleotide substitution, determined by the Shannon entropy, than non-progressors, which may implicate differences in selection to evade host immunity at these positions between the two groups.

In conclusion, irrespective of HBV genotypes, interindividual variability in the dynamics of viral load is not only associated with accumulation of HBV mutations that is increased in response to immune pressure, but differences in specific viral polymorphisms which differ between genotypes. Our population-based sequencing analysis incorporating long-term follow-up data of repeated measures of viral load and clinical variables has facilitated development of an analytical framework that links the dynamic process of hepatitis B progression and specific viral polymorphisms (or mutations) that could perhaps be incorporated into clinical testing.

## Supporting Information

Figure S1 **Comparisons in changing patterns of viral genetic diversity across the four phases of HBV-infection between overlapping and nonoverlapping reading frames.**
(PDF)

Figure S2 **Principal component analysis visualization for the cluster structure of viral load-associated viral SNPs with p values of ≤0.01.**
(PDF)

Figure S3 **The nucleotide sequence of the 18 subjects infected with HBV subgenotype Ba and 20 subjects infected with HBV subgenotype Ce who had deletion mutants.**
(PDF)

Table S1 **Primers and PCR conditions used for amplification and direct sequencing of the HBV polymerase region.**
(PDF)

Table S2 **Maximum likelihood fits of 24 different nucleotide substitution models.**
(PDF)

Table S3 **Baseline characteristics and follow-up in study population.**
(PDF)

Table S4 **Viral SNPs associated with longitudinal levels of viral load that are listed according to the sequence order of the polymerase region, stratified by HBV subgenotypes.**
(PDF)

Table S5 **Clusters of viral load-associated viral SNPs identified from principal component analysis, corresponding aa substitutions for these SNPs, and relationships of aa changes to sequence variation in overlapping or flanking T-cell epitopes, dynamics of viral load, and phase of chronic HBV infection.**
(PDF)

Table S6 **Shannon entropy values at each of the seven nucleotide positions in association with HCC development: comparison between participants with progression to HCC and non-progressors.**
(PDF)

Methods S1 **Direct sequencing, evaluation of nucleotide substitution models, and construction of consensus sequence.**
(PDF)

Results S1 **Cumulative incidences of HCC by phases of natural history of chronic hepatitis B in the subcohort.**
(PDF)
