# Supplementary material for: Impact of Genetic Heterogeneity in Polymerase of Hepatitis B Virus on Dynamics of Viral Load and Hepatitis B Progression
Source: PLoS One. 2013 Jul 30;8(7):e70169. doi: 10.1371/journal.pone.0070169 (PMC3728348; doi:10.1371/journal.pone.0070169)
Supplement: Figure S1 — Comparisons in changing patterns of viral genetic diversity across the four phases of HBV-infection between overlapping and nonoverlapping reading frames. (PDF) [file pone.0070169.s001.pdf]

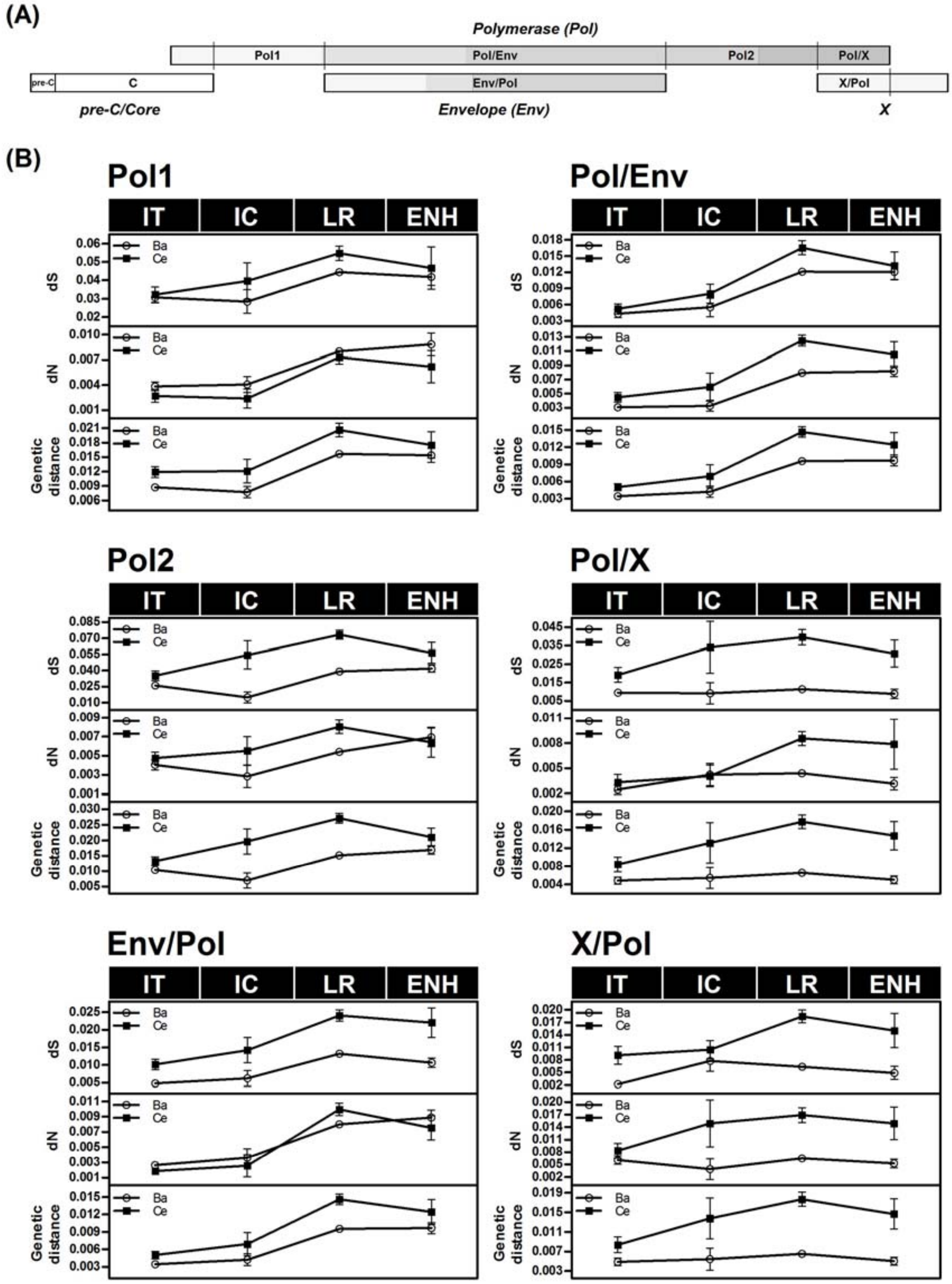

Figure S1. Comparisons in changing patterns of viral genetic diversity across the four phases of HBV-infection between overlapping and nonoverlapping reading frames. (A) The structure of the genes across a 2403-bp stretch in the sequence region. (B) Parameters of viral genetic diversity in different phases of HBV-infection. The polymerase region selected for analysis contains two nonoverlapping regions of the polymerase gene: nt2453-2847 and nt836-1373, and two overlapping open reading frames (ORFs): the largest overlapping ORF (1203-bp) of the HBV genome, which encodes polymerase and envelope, and a 250-bp overlapping ORF of polymerase and X.
