# Supplementary material for: Impact of Genetic Heterogeneity in Polymerase of Hepatitis B Virus on Dynamics of Viral Load and Hepatitis B Progression
Source: PLoS One. 2013 Jul 30;8(7):e70169. doi: 10.1371/journal.pone.0070169 (PMC3728348; doi:10.1371/journal.pone.0070169)
Supplement: Figure S2 — Principal component analysis visualization for the cluster structure of viral load-associated viral SNPs with p values of ≤0.01. (PDF) [file pone.0070169.s002.pdf]

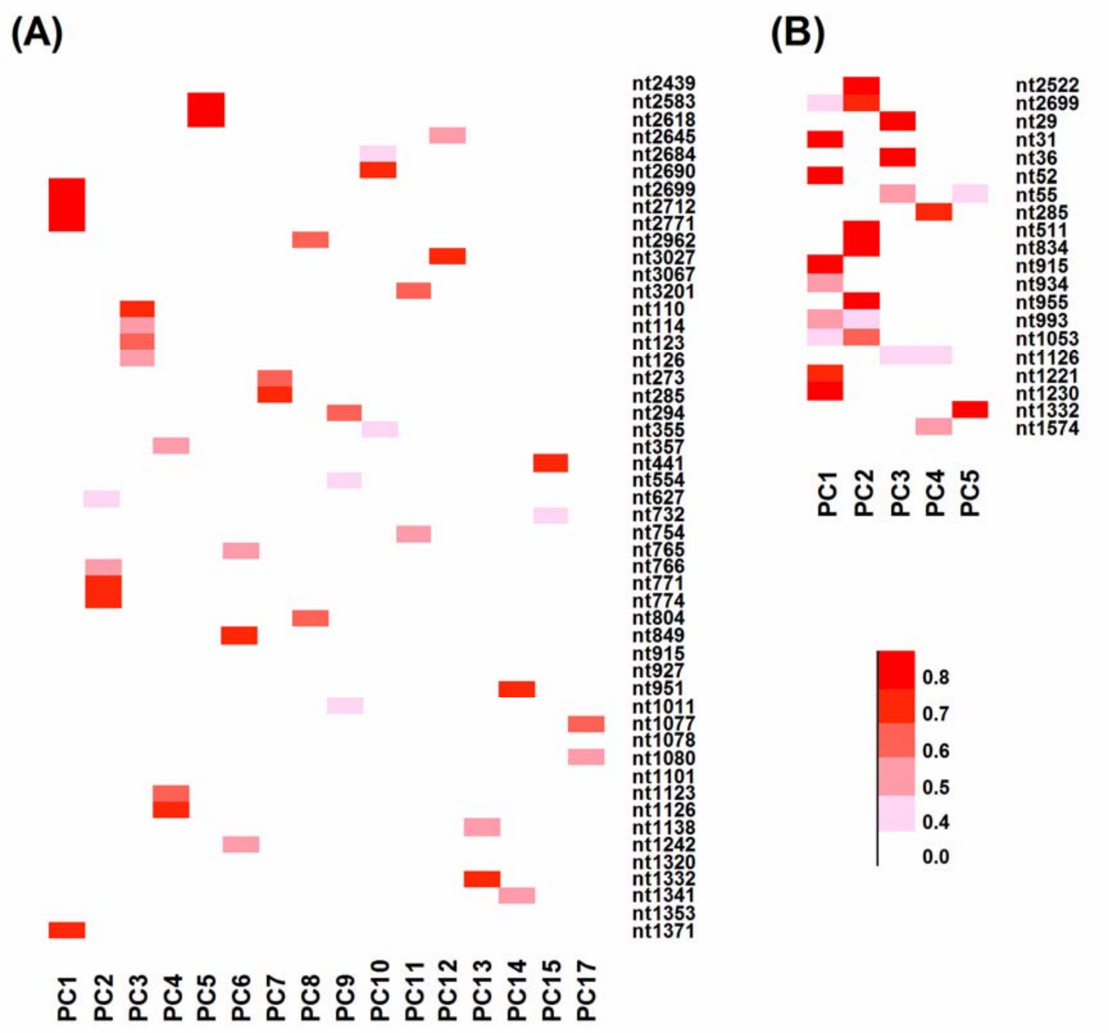

Figure S2. Principal component (PC) analysis visualization for the cluster structure of viral load-associated viral single nucleotide polymorphisms with p values of ≤0.01. Shown are top PCs with eigenvalues>1 that contain covarying viral SNPs in HBV subgenotype Ba (A) and Ce (B) group, respectively. The histogram bars are shown in color for factor-loading scores (a measure of the importance of the corresponding nucleotide variant type in defining particular PC). PC-correlated SNPs were those SNPs that shared ≥16% of the variance with the PC, corresponding to a factor-loading score (i.e., correlation) of ≥0.4.
