# Supplementary material for: Impact of Genetic Heterogeneity in Polymerase of Hepatitis B Virus on Dynamics of Viral Load and Hepatitis B Progression
Source: PLoS One. 2013 Jul 30;8(7):e70169. doi: 10.1371/journal.pone.0070169 (PMC3728348; doi:10.1371/journal.pone.0070169)
Supplement: Figure S3 — The nucleotide sequence of the 18 subjects infected with HBV subgenotype Ba and 20 subjects infected with HBV subgenotype Ce who had deletion mutants. (PDF) [file pone.0070169.s003.pdf]

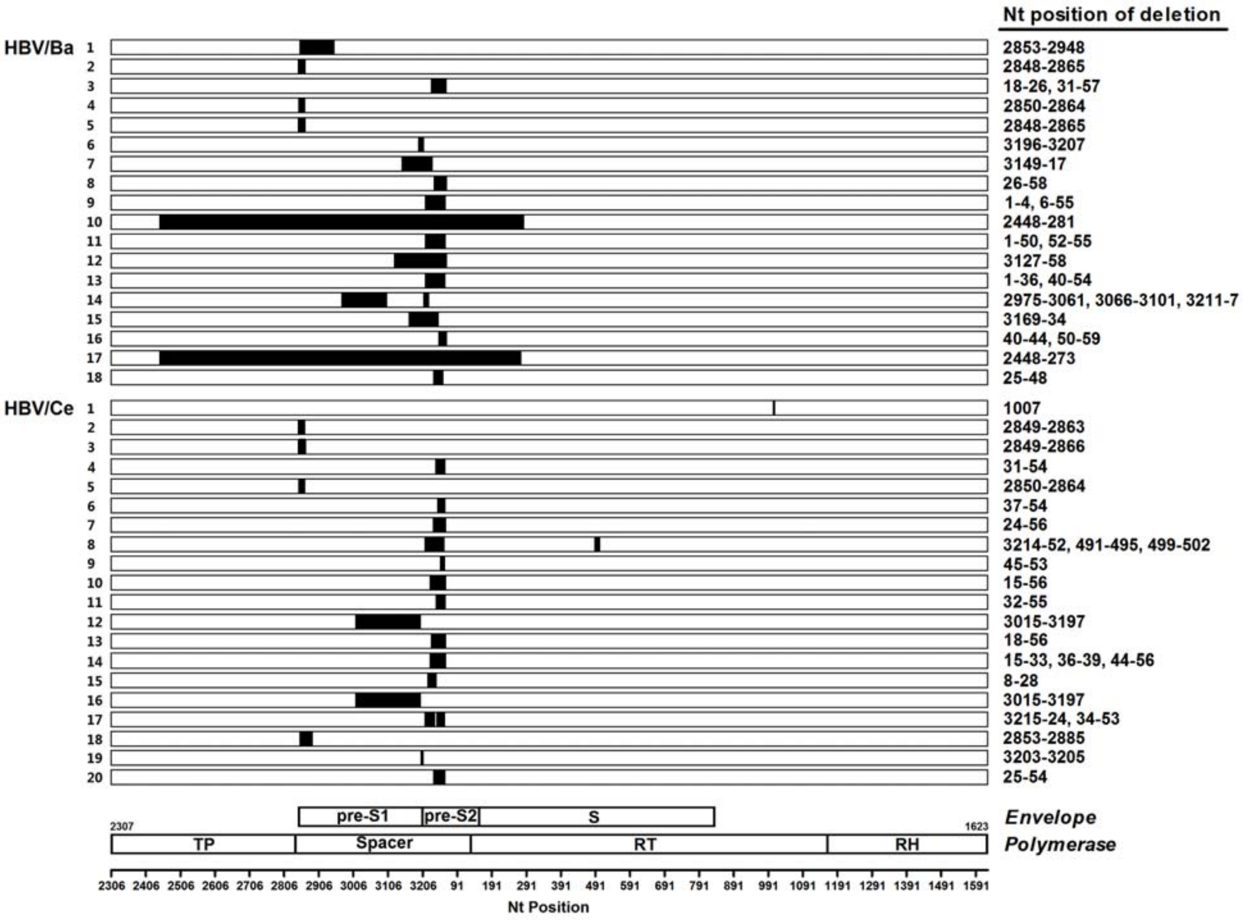


**Figure S3. The nucleotide (nt) sequence of the 18 subjects infected with HBV subgenotype Ba and 20 subjects infected with HBV subgenotype Ce who had deletion mutants.** A graphical map of the genes across the sequence region is shown at the bottom, and the number below the map indicates the nt site of the defined gene. Black box indicates the deleted region, and the number shown at right hand column indicates the nt position in alignment of the deletion. Deletions are clustered in pre-S1/pre-S2 region.
