# Supplementary material for: Impact of Genetic Heterogeneity in Polymerase of Hepatitis B Virus on Dynamics of Viral Load and Hepatitis B Progression
Source: PLoS One. 2013 Jul 30;8(7):e70169. doi: 10.1371/journal.pone.0070169 (PMC3728348; doi:10.1371/journal.pone.0070169)
Supplement: Table S1 — Primers and PCR conditions used for amplification and direct sequencing of the HBV polymerase region. (PDF) [file pone.0070169.s004.pdf]

Table S1. Primers and PCR conditions used for amplification and direct sequencing of the HBV polymerase region.

First-round PCR	
PCR condition: initial denaturation at 94℃ 4 min, followed by 34 cycles of amplification consisting of denaturation at 94℃ 40 sec, annealing at 55℃ 30 sec, and extension at 72℃ 1.5 min, then a final extension at 72℃ 10 min	
NP1 pair (full-length PCR)	
P1 (Forward, nt1824-1843)	5'-TTCACCTCTGCCTAATCATC-3'
P2 (Reverse, nt1803-1784)	5'-AACAGACCAATTTATGCCTA-3'
NP2 pair	
P1 (Forward, nt1824-1843)	5'-TTCACCTCTGCCTAATCATC-3'
P4 (Reverse, nt734-715)	5'-CTGAAAGCCAAACAGTGGGG-3'
NP3 pair	
P2 (Reverse, nt1803-1784)	5'-AACAGACCAATTTATGCCTA-3'
P3 (Forward, nt3193-3213)	5'-CATCCTCAGGCCATGCAGTGG-3'
Second-round PCR	
PCR condition: initial denaturation at 96℃ 3 min, followed by 10 cycles of amplification at 94℃ 1 min, 60℃ 30 sec, and 72℃ 1.5 min, then 40 cycles of amplification at 94℃ 1 min, 56℃ 30 sec, and 72℃ 1.5 min, then a final extension at 72℃ 10 min	
NP4 pair	
P5 (Forward, nt2300-2325)	5'-CCACCAAATGCCCCTATCTTATCAAC-3'
P6 (Reverse, nt479-462)	5'-GAGGACAAACGGGCAACA-3'
NP5 pair	
P7 (Forward, nt353-373)	5'-CCAATTTGTCCTGGCTATCGC-3'
P8 (Reverse, nt1718-1694)	5'-ACAGTCTTTGAAGTAKGCCTCAAGG-3'
Sequencing reaction	
P5 (Forward, nt2300-2325)	5'-CCACCAAATGCCCCTATCTTATCAAC-3'
S1 (Forward, nt2512-2531)	5'-TCTTTAATCCTGAATGGCAA-3'
S2 (Forward, nt2816-2835)	5'-GTCACCATATTCTTGGGAAC-3'
P3 (Forward, nt3193-3213)	5'-CATCCTCAGGCCATGCAGTGG-3'
P6 (Reverse, nt479-462)	5'-GAGGACAAACGGGCAACA-3'
P7 (Forward, nt353-373)	5'-CCAATTTGTCCTGGCTATCGC-3'
S3 (Forward, nt752-771)	5'-TATTGGGGGCCAAGTCTGTA-3'
S4 (Forward, nt1142-1159)	5'-TTTACCCCGTTGCYCGGC-3'
P8 (Reverse, nt1718-1694)	5'-ACAGTCTTTGAAGTAKGCCTCAAGG-3'

HBV, hepatitis B virus; PCR, polymerase chain reaction.
