# Supplementary material for: Impact of Genetic Heterogeneity in Polymerase of Hepatitis B Virus on Dynamics of Viral Load and Hepatitis B Progression
Source: PLoS One. 2013 Jul 30;8(7):e70169. doi: 10.1371/journal.pone.0070169 (PMC3728348; doi:10.1371/journal.pone.0070169)
Supplement: Table S2 — Maximum likelihood fits of 24 different nucleotide substitution models. (PDF) [file pone.0070169.s005.pdf]

Table S2. Maximum likelihood fits of 24 different nucleotide substitution models.

Model	Parameters	BIC	AICc	*lnL*	(+*I*)	(+*G*)	*R*	*f*(A)	*f*(T)	*f*(C)	*f*(G)	*r*(AT)	*r*(AC)	*r*(AG)	*r*(TA)	*r*(TC)	*r*(TG)	*r*(CA)	*r*(CT)	*r*(CG)	*r*(GA)	*r*(GT)	*r*(GC)
GTR+G+I	1157	140582.072	126541.918	-62112.986	0.44	0.68	1.47	0.221	0.277	0.278	0.220	0.050	0.110	0.163	0.040	0.134	0.036	0.088	0.134	0.016	0.164	0.045	0.020
GTR+G	1156	141176.338	127148.318	-62417.188	n/a	0.31	1.48	0.221	0.277	0.278	0.220	0.050	0.109	0.167	0.040	0.131	0.036	0.087	0.131	0.016	0.168	0.045	0.020
K2+G+I	1150	141718.362	127763.141	-62730.610	0.43	0.66	1.83	0.250	0.250	0.250	0.250	0.044	0.044	0.162	0.044	0.162	0.044	0.044	0.162	0.044	0.162	0.044	0.044
T92+G+I	1151	141726.442	127759.088	-62727.581	0.43	0.66	1.83	0.251	0.251	0.249	0.249	0.044	0.044	0.161	0.044	0.161	0.044	0.044	0.162	0.044	0.162	0.044	0.044
K2+G	1149	142348.612	128405.524	-63052.803	n/a	0.32	1.83	0.250	0.250	0.250	0.250	0.044	0.044	0.162	0.044	0.162	0.044	0.044	0.162	0.044	0.162	0.044	0.044
T92+G	1150	142357.523	128402.302	-63050.190	n/a	0.32	1.83	0.251	0.251	0.249	0.249	0.044	0.044	0.161	0.044	0.161	0.044	0.044	0.162	0.044	0.162	0.044	0.044
JC+G+I	1149	144279.144	130336.057	-64018.069	0.44	0.67	0.50	0.250	0.250	0.250	0.250	0.083	0.083	0.083	0.083	0.083	0.083	0.083	0.083	0.083	0.083	0.083	0.083
JC+G	1148	144905.061	130974.106	-64338.096	n/a	0.32	0.50	0.250	0.250	0.250	0.250	0.083	0.083	0.083	0.083	0.083	0.083	0.083	0.083	0.083	0.083	0.083	0.083
GTR+I	1156	147240.978	133212.957	-65449.508	0.51	n/a	1.28	0.221	0.277	0.278	0.220	0.050	0.100	0.138	0.040	0.141	0.041	0.080	0.140	0.036	0.139	0.051	0.045
K2+I	1149	147991.802	134048.714	-65874.398	0.51	n/a	1.75	0.250	0.250	0.250	0.250	0.045	0.045	0.159	0.045	0.159	0.045	0.045	0.159	0.045	0.159	0.045	0.045
T92+I	1150	148000.795	134045.575	-65871.826	0.51	n/a	1.75	0.251	0.251	0.249	0.249	0.046	0.045	0.158	0.046	0.158	0.045	0.046	0.160	0.045	0.160	0.046	0.045
JC+I	1148	150534.532	136603.578	-67152.832	0.51	n/a	0.50	0.250	0.250	0.250	0.250	0.083	0.083	0.083	0.083	0.083	0.083	0.083	0.083	0.083	0.083	0.083	0.083
GTR	1155	157855.765	143839.878	-70763.970	n/a	n/a	1.20	0.221	0.277	0.278	0.220	0.055	0.109	0.137	0.044	0.134	0.037	0.087	0.134	0.036	0.138	0.046	0.045
K2	1148	158868.108	144937.153	-71319.619	n/a	n/a	1.75	0.250	0.250	0.250	0.250	0.045	0.045	0.159	0.045	0.159	0.045	0.045	0.159	0.045	0.159	0.045	0.045
T92	1149	158881.613	144938.525	-71319.303	n/a	n/a	1.75	0.251	0.251	0.249	0.249	0.046	0.045	0.158	0.046	0.158	0.045	0.046	0.160	0.045	0.160	0.046	0.045
JC	1147	161384.801	147465.980	-72585.034	n/a	n/a	0.50	0.250	0.250	0.250	0.250	0.083	0.083	0.083	0.083	0.083	0.083	0.083	0.083	0.083	0.083	0.083	0.083
HKY+I	1152	2825207.256	2811227.769	-1404460.920	0.07	n/a	1.85	0.221	0.277	0.278	0.220	0.050	0.050	0.142	0.040	0.179	0.039	0.040	0.179	0.039	0.143	0.050	0.050
HKY	1151	2987001.979	2973034.625	-1485365.350	n/a	n/a	1.84	0.221	0.277	0.278	0.220	0.050	0.050	0.142	0.040	0.179	0.040	0.040	0.179	0.040	0.142	0.050	0.050
TN93+I	1153	3506507.401	3492515.780	-1745103.924	0.02	n/a	1.67	0.221	0.277	0.278	0.220	0.052	0.052	0.190	0.041	0.124	0.041	0.041	0.123	0.041	0.191	0.052	0.052
TN93	1152	3642629.234	3628649.746	-1813171.909	n/a	n/a	1.79	0.221	0.277	0.278	0.220	0.050	0.050	0.184	0.040	0.137	0.040	0.040	0.137	0.040	0.185	0.050	0.050
TN93+G	1153	5683533.062	5669541.441	-2833616.755	n/a	0.26	1.50	0.221	0.277	0.278	0.220	0.056	0.056	0.168	0.044	0.132	0.044	0.044	0.132	0.044	0.169	0.056	0.056
TN93+G+I	1154	5683589.441	5669585.687	-2833637.876	0.00	0.26	1.48	0.221	0.277	0.278	0.220	0.056	0.056	0.166	0.045	0.133	0.044	0.045	0.132	0.044	0.166	0.056	0.056
HKY+G+I	1153	5729674.133	5715682.513	-2856687.290	0.00	0.24	1.88	0.221	0.277	0.278	0.220	0.049	0.049	0.143	0.039	0.180	0.039	0.039	0.180	0.039	0.143	0.049	0.049
HKY+G	1152	5752223.473	5738243.985	-2867969.028	n/a	0.24	1.88	0.221	0.277	0.278	0.220	0.049	0.049	0.143	0.039	0.180	0.039	0.039	0.180	0.039	0.143	0.049	0.049

Abbreviations: AICc; corrected Akaike information criterion; BIC, Bayesian information criterion; GTR: general time reversible; HKY: Hasegawa-Kishino-Yano; *lnL*: maximum likelihood; TN93: Tamura-Nei; T92: Tamura 3-parameter; K2: Kimura 2-parameter; JC: Jukes-Cantor.

Note: Non-uniformity of evolutionary rates among sites may be modeled by using a discrete Gamma distribution (+*G*) with 5 rate categories and by assuming that a certain fraction of sites are evolutionarily invariable (+*I*). Whenever applicable, estimates of gamma shape parameter and/or the estimated fraction of invariant sites are shown. Assumed or estimated values of transition/transversion bias (*R*) are shown for each model, as well. They are followed by nucleotide frequencies (*f*) and rates of base substitutions (*r*) for each nucleotide pair. Relative values of instantaneous *r* should be considered when evaluating them. For simplicity, sum of *r* values is made equal to 1 for each model. For estimating maximum likelihood values, a tree topology was automatically computed. All positions with less than 95% site coverage were eliminated. That is, fewer than 5% alignment gaps, missing data, and ambiguous bases were allowed at any position.
