# Supplementary material for: Impact of Genetic Heterogeneity in Polymerase of Hepatitis B Virus on Dynamics of Viral Load and Hepatitis B Progression
Source: PLoS One. 2013 Jul 30;8(7):e70169. doi: 10.1371/journal.pone.0070169 (PMC3728348; doi:10.1371/journal.pone.0070169)
Supplement: Table S3 — Baseline characteristics and follow-up in study population. (PDF) [file pone.0070169.s006.pdf]

Table S3. Baseline characteristics and follow-up in study population[a].

	Total	Natural history of chronic HBV infection[b]			
		IT	IC	LR	ENH
**Entire sample, n**	1143	86	23	932	71
Cigarette smoking, n (%)	356 (31.2)	27 (31.4)	8 (34.8)	294 (31.6)	21 (29.6)
Alcohol consumption, n (%)	239 (20.9)	12 (14.0)	6 (26.1)	199 (21.4)	13 (18.3)
Body mass index, mean±SD, kg/m^2[c]	23.8±2.6	23.9±2.4	23.3±2.7	23.7±2.6	25.0± 2.6
Elevated ALT≥1 visit, n (%)[c]	423 (41.2)	55 (72.4)	16 (76.2)	305 (36.1)	38 (65.5)
Elevated AST≥1 visit, n (%)[c]	317 (30.9)	45 (59.2)	13 (61.9)	223 (26.4)	30 (51.7)
Trajectory patterns of viral load during follow-up[c,d]					
Sustained low, n (%)	686 (60.0)	8 (9.3)	4 (17.4)	621 (66.6)	34 (47.9)
Sustained high, n (%)	367 (32.1)	16 (18.6)	6 (26.1)	299 (32.1)	34 (47.9)
Extremely high to low, n (%)	90 (7.9)	62 (72.1)	13 (56.5)	12 (1.3)	3 (4.2)
**Inclusion for sequence analysis**					
Plasma HBV DNA≥1000 copies/mL, n	867	83	23	674	59
Successful sequencing, n	575	64	13	443	36
Cigarette smoking, n (%)	180 (31.3)	20 (31.3)	4 (30.8)	140 (31.6)	12 (33.3)
Alcohol consumption, n (%)	125 (21.7)	8 (12.5)	3 (23.1)	102 (23.0)	6 (16.7)
Body mass index, mean±SD, kg/m^2[c]	23.6±2.7	23.7±2.4	23.4±2.3	23.5±2.7	24.8±2.7
Elevated ALT≥1 visit, n (%)[c]	235 (46.3)	40 (72.7)	10 (90.9)	163 (40.8)	14 (51.9)
Elevated AST≥1 visit, n (%)[c]	183 (36.0)	35 (63.6)	8 (72.7)	121 (30.3)	14 (51.9)
Trajectory patterns of viral load during follow-up[c,d]					
Sustained low, n (%)	251 (43.7)	4 (6.3)	1 (7.7)	223 (50.3)	13 (36.1)
Sustained high, n (%)	252 (43.8)	7 (10.9)	3 (23.1)	213 (48.1)	20 (55.6)
Extremely high to low, n (%)	72 (12.5)	53 (82.8)	9 (69.2)	7 (1.6)	3 (8.3)

ALT, alanine aminotransferase; AST, aspartate aminotransferase; ENH, HBeAg negative hepatitis; HBV, hepatitis B virus; IT, immune-tolerant; IC, immune-clearance; LR, non/low-replicative.

[a]Total number of subjects may vary because of missing value due to PCR/sequencing failure or nonparticipation in follow-up examination.

[b]31 subjects with missing data on baseline ALT levels or HBeAg status were excluded from analysis of natural history.

[c]$P<0.05$ between the four phases of natural history determined with Kruskall-Wallis test (continuous variable) or χ^2 test (categorical variable).

[d]Three patterns of time trend for viral load were defined based on 7706 serial samples collected from 1143 subjects during 16 years of follow-up according to our previous longitudinal viral-load study [reference 13].
