# Supplementary material for: Impact of Genetic Heterogeneity in Polymerase of Hepatitis B Virus on Dynamics of Viral Load and Hepatitis B Progression
Source: PLoS One. 2013 Jul 30;8(7):e70169. doi: 10.1371/journal.pone.0070169 (PMC3728348; doi:10.1371/journal.pone.0070169)
Supplement: Table S4 — Viral SNPs associated with longitudinal levels of viral load that are listed according to the sequence order of the polymerase region, stratified by HBV subgenotypes. (PDF) [file pone.0070169.s007.pdf]

Table S4. Viral single nucleotide polymorphisms (SNPs) associated with longitudinal levels of viral load that are listed according to the sequence order of the polymerase region, stratified by HBV subgenotypes.

Nucleotide (nt) position	Freq. of variant SNP	β	95% CI	P	Nucleotide (nt) position	Freq. of variant SNP	β	95% CI	P	Nucleotide (nt) position	Freq. of variant SNP	β	95% CI	P
**Entire, subgenotype Ba**					nt287	1.74	-0.81	(-1.61,-0.01)	0.0473	nt1080	19.13	-0.56	(-0.82,-0.29)	<0.0001
nt2439	8.91	-0.59	(-0.96,-0.22)	0.0019	nt294	8.26	-0.78	(-1.16,-0.40)	0.0001	nt1101	1.74	-1.19	(-1.95,-0.43)	0.0022
nt2441	24.78	-0.31	(-0.56,-0.07)	0.0120	nt327	2.17	-0.81	(-1.56,-0.06)	0.0351	nt1104	3.70	-0.69	(-1.24,-0.14)	0.0147
nt2452	6.09	-0.48	(-0.93,-0.02)	0.0393	nt330	6.30	-0.52	(-0.96,-0.07)	0.0229	nt1113	2.17	0.76	(0.03,1.49)	0.0419
nt2507	1.74	-0.88	(-1.74,-0.03)	0.0429	nt355	4.78	-0.72	(-1.21,-0.23)	0.0042	nt1123	38.70	-0.43	(-0.65,-0.21)	0.0001
nt2549	6.74	0.55	(0.12,0.97)	0.0113	nt357	9.57	-0.74	(-1.10,-0.37)	0.0001	nt1126	21.52	-0.61	(-0.87,-0.35)	<0.0001
nt2583	29.13	-0.36	(-0.59,-0.12)	0.0028	nt358	1.30	-0.98	(-1.87,-0.09)	0.0305	nt1138	8.91	-0.79	(-1.16,-0.42)	<0.0001
nt2617	13.70	-0.31	(-0.62,-0.00)	0.0483	nt390	2.39	-0.85	(-1.50,-0.19)	0.0115	nt1139	4.57	-0.52	(-1.02,-0.02)	0.0419
nt2618	24.35	-0.34	(-0.59,-0.09)	0.0070	nt400	15.22	-0.32	(-0.61,-0.03)	0.0312	nt1165	61.96	0.29	(0.07,0.50)	0.0103
nt2645	2.61	1.19	(0.43,1.95)	0.0023	nt408	4.35	-0.68	(-1.22,-0.14)	0.0131	nt1242	19.35	-0.37	(-0.64,-0.10)	0.0071
nt2659	1.30	1.06	(0.07,2.04)	0.0362	nt429	1.30	-1.02	(-1.98,-0.07)	0.0357	nt1290	5.22	-0.52	(-0.99,-0.05)	0.0286
nt2678	5.43	-0.55	(-1.03,-0.07)	0.0258	nt436	1.74	-0.94	(-1.70,-0.18)	0.0149	nt1320	18.04	-0.38	(-0.66,-0.10)	0.0070
nt2684	6.09	-0.65	(-1.09,-0.21)	0.0036	nt438	1.74	-1.09	(-1.92,-0.26)	0.0105	nt1332	2.83	-0.90	(-1.53,-0.27)	0.0054
nt2690	8.48	-0.51	(-0.90,-0.13)	0.0089	nt441	1.74	-1.10	(-1.90,-0.31)	0.0063	nt1341	3.26	-0.86	(-1.45,-0.27)	0.0044
nt2699	53.70	-0.29	(-0.50,-0.07)	0.0085	nt533	3.26	-0.74	(-1.33,-0.14)	0.0151	nt1353	1.74	-1.39	(-2.15,-0.62)	0.0004
nt2712	43.70	0.39	(0.18,0.60)	0.0003	nt551	3.91	-0.58	(-1.14,-0.01)	0.0446	nt1359	3.91	-0.60	(-1.14,-0.06)	0.0296
nt2739	51.74	0.25	(0.03,0.46)	0.0238	nt554	1.74	-1.13	(-1.93,-0.33)	0.0056	nt1368	28.70	-0.29	(-0.52,-0.05)	0.0167
nt2753	4.13	-0.57	(-1.11,-0.03)	0.0386	nt624	1.30	-0.98	(-1.96,-0.00)	0.0500	nt1371	27.39	-0.41	(-0.64,-0.17)	0.0008
nt2771	51.74	-0.28	(-0.49,-0.07)	0.0101	nt627	2.83	-0.88	(-1.53,-0.22)	0.0085	nt1407	6.30	-0.54	(-0.97,-0.10)	0.0161
nt2792	3.04	-0.66	(-1.31,-0.01)	0.0461	nt705	4.78	-0.58	(-1.10,-0.06)	0.0283	nt1509	3.91	-0.69	(-1.22,-0.16)	0.0114
nt2962	5.87	-0.73	(-1.19,-0.28)	0.0015	nt732	3.04	-1.01	(-1.60,-0.41)	0.0009					
nt3027	5.43	0.85	(0.39,1.32)	0.0003	nt753	53.26	0.26	(0.04,0.47)	0.0191	**Entire, subgenotype Ce**				
nt3067	9.13	0.66	(0.30,1.03)	0.0004	nt754	1.52	-1.13	(-1.98,-0.28)	0.0092	nt2441	12.63	-1.42	(-2.59,-0.25)	0.0177
nt3097	51.30	0.26	(0.04,0.47)	0.0185	nt762	1.74	-0.96	(-1.73,-0.19)	0.0143	nt2443	13.68	-1.30	(-2.44,-0.15)	0.0273
nt3153	2.83	-0.80	(-1.45,-0.15)	0.0155	nt765	19.57	-0.45	(-0.72,-0.18)	0.0011	nt2522	28.42	1.25	(0.39,2.11)	0.0049
nt3157	4.35	-0.62	(-1.17,-0.08)	0.0255	nt766	7.61	-0.52	(-0.92,-0.12)	0.0104	nt2574	73.68	0.93	(0.04,1.83)	0.0404
nt3158	2.61	-0.84	(-1.52,-0.16)	0.0160	nt771	4.78	-1.41	(-1.89,-0.92)	<0.0001	nt2608	7.37	-1.77	(-3.21,-0.34)	0.0160
nt3192	8.26	-0.39	(-0.77,-0.01)	0.0466	nt774	6.09	-1.05	(-1.48,-0.62)	<0.0001	nt2699	55.79	-1.40	(-2.17,-0.63)	0.0005
nt3195	1.74	0.89	(0.03,1.76)	0.0425	nt802	1.30	-0.91	(-1.82,-0.00)	0.0498	nt2898	11.58	-1.39	(-2.59,-0.19)	0.0240
nt3201	8.26	-0.54	(-0.94,-0.14)	0.0079	nt804	1.52	-1.29	(-2.15,-0.44)	0.0032	nt2946	93.68	1.69	(0.12,3.26)	0.0357
nt3211	2.39	0.73	(0.02,1.43)	0.0441	nt813	1.74	-0.97	(-1.77,-0.18)	0.0168	nt3084	7.37	-1.60	(-3.10,-0.09)	0.0382
nt3213	1.52	1.13	(0.19,2.06)	0.0182	nt849	5.87	-0.78	(-1.23,-0.34)	0.0006	nt3116	31.58	-0.95	(-1.81,-0.10)	0.0291
nt11	3.48	0.69	(0.10,1.28)	0.0214	nt915	15.00	0.40	(0.10,0.69)	0.0091	nt16	9.47	-1.58	(-2.91,-0.25)	0.0200
nt23	1.74	0.93	(0.03,1.83)	0.0437	nt925	3.26	-0.64	(-1.22,-0.05)	0.0332	nt28	9.47	-1.67	(-3.01,-0.33)	0.0150
nt27	3.04	0.83	(0.15,1.50)	0.0162	nt927	3.26	-1.00	(-1.59,-0.40)	0.0010	nt29	10.53	-1.77	(-3.01,-0.52)	0.0059
nt110	6.74	-0.74	(-1.18,-0.31)	0.0009	nt941	2.61	-0.77	(-1.46,-0.07)	0.0312	nt30	6.32	-1.69	(-3.28,-0.09)	0.0384
nt114	6.74	-0.69	(-1.12,-0.26)	0.0016	nt951	19.13	-0.38	(-0.65,-0.11)	0.0056	nt31	35.79	-1.38	(-2.18,-0.58)	0.0009
nt123	8.04	-0.73	(-1.12,-0.33)	0.0003	nt987	13.26	-0.38	(-0.70,-0.06)	0.0192	nt32	8.42	-1.72	(-3.13,-0.31)	0.0175
nt126	14.57	-0.43	(-0.73,-0.12)	0.0057	nt1005	1.30	-1.10	(-2.03,-0.17)	0.0205	nt33	8.42	-1.72	(-3.13,-0.31)	0.0175
nt154	2.61	-0.69	(-1.35,-0.03)	0.0406	nt1011	3.26	-0.79	(-1.37,-0.21)	0.0075	nt34	8.42	-1.49	(-2.92,-0.06)	0.0414
nt219	2.39	-0.88	(-1.61,-0.15)	0.0181	nt1017	1.96	-0.99	(-1.75,-0.23)	0.0107	nt35	8.42	-1.49	(-2.92,-0.06)	0.0414
nt255	3.26	-0.78	(-1.38,-0.17)	0.0122	nt1029	14.35	-0.32	(-0.62,-0.03)	0.0335	nt36	15.79	-1.57	(-2.64,-0.50)	0.0046
nt273	39.78	-0.44	(-0.66,-0.23)	0.0001	nt1077	3.04	-0.95	(-1.57,-0.33)	0.0028	nt37	10.53	-1.34	(-2.62,-0.07)	0.0387
nt285	35.43	-0.45	(-0.67,-0.23)	0.0001	nt1078	2.39	1.29	(0.59,1.99)	0.0003	nt38	10.53	-1.34	(-2.62,-0.07)	0.0387

Nucleotide (nt) position	Freq. of variant SNP	β	95% CI	P	Nucleotide (nt) position	Freq. of variant SNP	β	95% CI	P	Nucleotide (nt) position	Freq. of variant SNP	β	95% CI	P
nt39	11.58	-1.28	(-2.50,-0.05)	0.0418	nt2708	4.03	0.52	(0.05,0.99)	0.0293	nt355	5.54	-0.58	(-0.95,-0.20)	0.0025
nt41	10.53	-1.35	(-2.63,-0.07)	0.0384	nt2712	41.06	0.18	(0.00,0.36)	0.0439	nt357	9.82	-0.59	(-0.88,-0.29)	0.0001
nt42	13.68	-1.18	(-2.33,-0.03)	0.0440	nt2721	7.56	0.39	(0.06,0.73)	0.0204	nt358	1.51	-0.75	(-1.43,-0.08)	0.0284
nt44	14.74	-1.23	(-2.34,-0.13)	0.0294	nt2733	2.27	-0.60	(-1.19,-0.01)	0.0472	nt381	17.88	0.35	(0.12,0.57)	0.0024
nt47	11.58	-1.39	(-2.60,-0.17)	0.0258	nt2753	4.28	-0.63	(-1.05,-0.20)	0.0038	nt390	2.77	-0.59	(-1.09,-0.09)	0.0209
nt48	13.68	-1.40	(-2.53,-0.28)	0.0153	nt2763	1.76	0.77	(0.13,1.41)	0.0186	nt408	5.04	-0.55	(-0.96,-0.14)	0.0093
nt50	11.58	-1.39	(-2.60,-0.17)	0.0258	nt2790	2.52	-0.60	(-1.18,-0.02)	0.0433	nt429	1.51	-0.93	(-1.66,-0.21)	0.0121
nt51	11.58	-1.39	(-2.60,-0.17)	0.0258	nt2837	4.53	0.48	(0.05,0.91)	0.0302	nt436	1.76	-0.63	(-1.22,-0.03)	0.0396
nt52	69.47	1.12	(0.27,1.97)	0.0101	nt2860	4.79	0.46	(0.05,0.87)	0.0288	nt438	2.02	-0.92	(-1.56,-0.29)	0.0044
nt55	11.58	-1.60	(-2.84,-0.36)	0.0117	nt2892	1.51	0.91	(0.09,1.73)	0.0297	nt441	2.02	-0.98	(-1.58,-0.38)	0.0015
nt285	14.74	-1.41	(-2.50,-0.32)	0.0118	nt2944	4.79	0.45	(0.03,0.88)	0.0373	nt533	3.53	-0.54	(-1.01,-0.08)	0.0216
nt393	6.32	-1.77	(-3.38,-0.16)	0.0313	nt2945	4.79	0.45	(0.03,0.88)	0.0373	nt554	1.76	-0.84	(-1.50,-0.18)	0.0130
nt511	35.79	1.39	(0.58,2.19)	0.0009	nt2962	6.05	-0.53	(-0.89,-0.16)	0.0045	nt624	1.51	-0.79	(-1.54,-0.03)	0.0406
nt796	80.00	1.08	(0.10,2.06)	0.0304	nt2986	3.78	0.73	(0.26,1.19)	0.0022	nt627	3.27	-0.75	(-1.25,-0.25)	0.0031
nt834	60.00	-1.20	(-1.99,-0.40)	0.0035	nt2993	1.51	1.08	(0.29,1.88)	0.0075	nt678	1.76	-0.68	(-1.32,-0.04)	0.0370
nt841	91.58	1.64	(0.28,3.00)	0.0188	nt2997	2.02	0.76	(0.09,1.43)	0.0269	nt705	4.79	-0.42	(-0.84,-0.01)	0.0465
nt915	32.63	-1.17	(-2.02,-0.33)	0.0067	nt3004	1.76	0.71	(0.04,1.38)	0.0388	nt706	1.51	1.22	(0.48,1.96)	0.0014
nt934	54.74	1.83	(1.09,2.57)	<0.0001	nt3013	3.27	0.79	(0.28,1.29)	0.0024	nt732	3.53	-0.88	(-1.33,-0.43)	0.0001
nt951	90.53	-1.61	(-2.99,-0.23)	0.0227	nt3018	1.76	0.78	(0.06,1.50)	0.0344	nt754	1.51	-1.12	(-1.83,-0.40)	0.0022
nt955	38.95	1.38	(0.59,2.17)	0.0008	nt3026	4.28	0.49	(0.05,0.93)	0.0304	nt762	1.76	-0.91	(-1.54,-0.28)	0.0045
nt993	31.58	-1.12	(-1.97,-0.27)	0.0106	nt3027	5.54	0.71	(0.33,1.09)	0.0003	nt766	8.31	-0.36	(-0.67,-0.05)	0.0240
nt1053	54.74	1.20	(0.42,1.98)	0.0028	nt3031	1.76	0.78	(0.00,1.55)	0.0494	nt771	5.54	-1.30	(-1.67,-0.93)	<0.0001
nt1126	8.42	-1.85	(-3.22,-0.48)	0.0086	nt3067	9.32	0.48	(0.18,0.78)	0.0017	nt774	6.55	-0.92	(-1.26,-0.58)	<0.0001
nt1218	33.68	-1.09	(-1.94,-0.24)	0.0123	nt3150	4.03	0.47	(0.01,0.93)	0.0432	nt802	1.51	-0.70	(-1.39,-0.01)	0.0476
nt1221	42.11	-1.44	(-2.22,-0.66)	0.0004	nt3153	3.27	-0.69	(-1.19,-0.19)	0.0067	nt804	1.76	-1.06	(-1.72,-0.40)	0.0016
nt1230	30.53	-1.31	(-2.16,-0.47)	0.0027	nt3157	4.79	-0.47	(-0.89,-0.04)	0.0317	nt813	2.02	-0.88	(-1.48,-0.28)	0.0044
nt1323	78.95	1.06	(0.10,2.02)	0.0308	nt3158	2.77	-0.73	(-1.26,-0.19)	0.0080	nt831	2.52	-0.64	(-1.21,-0.08)	0.0263
nt1332	90.53	1.71	(0.42,2.99)	0.0097	nt3201	9.07	-0.51	(-0.83,-0.20)	0.0015	nt849	6.55	-0.61	(-0.95,-0.27)	0.0005
nt1479	62.11	-0.82	(-1.64,-0.01)	0.0483	nt3211	2.52	0.65	(0.08,1.22)	0.0257	nt897	2.02	-0.66	(-1.27,-0.04)	0.0356
nt1485	25.26	-1.00	(-1.92,-0.08)	0.0339	nt3213	1.51	0.82	(0.03,1.61)	0.0419	nt904	3.02	-0.75	(-1.26,-0.25)	0.0037
nt1574	25.26	-1.90	(-2.74,-1.07)	<0.0001	nt1	10.58	0.34	(0.05,0.63)	0.0223	nt915	14.11	0.34	(0.09,0.59)	0.0078
					nt27	3.02	0.55	(-0.02,1.11)	0.0588	nt927	3.53	-0.70	(-1.17,-0.23)	0.0034
**HBeAg-negative, subgenotype Ba**					nt46	5.79	-0.39	(-0.77,-0.00)	0.0492	nt941	2.77	-0.61	(-1.17,-0.04)	0.0353
nt2439	9.57	-0.36	(-0.66,-0.07)	0.0155	nt53	31.99	0.20	(0.02,0.39)	0.0309	nt950	3.02	-0.63	(-1.14,-0.11)	0.0170
nt2452	6.55	-0.41	(-0.77,-0.05)	0.0275	nt110	6.80	-0.70	(-1.06,-0.34)	0.0001	nt1005	1.51	-0.97	(-1.68,-0.26)	0.0075
nt2507	2.02	-0.74	(-1.39,-0.08)	0.0272	nt114	6.30	-0.59	(-0.95,-0.23)	0.0014	nt1008	2.52	0.76	(0.18,1.34)	0.0102
nt2511	3.53	-0.51	(-1.01,-0.01)	0.0472	nt123	8.82	-0.48	(-0.79,-0.17)	0.0024	nt1011	3.53	-0.79	(-1.25,-0.33)	0.0008
nt2549	6.80	0.52	(0.17,0.87)	0.0040	nt126	15.62	-0.25	(-0.49,-0.01)	0.0404	nt1017	2.27	-0.71	(-1.30,-0.13)	0.0163
nt2559	11.08	-0.32	(-0.60,-0.04)	0.0241	nt166	3.53	-0.51	(-0.99,-0.04)	0.0332	nt1059	2.77	-0.58	(-1.11,-0.05)	0.0320
nt2564	2.27	0.75	(0.07,1.42)	0.0301	nt219	2.52	-0.74	(-1.33,-0.15)	0.0148	nt1077	3.53	-0.68	(-1.15,-0.20)	0.0054
nt2603	3.78	-0.66	(-1.11,-0.20)	0.0048	nt246	2.27	-0.59	(-1.18,-0.01)	0.0458	nt1078	1.76	0.83	(0.16,1.50)	0.0148
nt2645	2.02	0.89	(0.14,1.65)	0.0200	nt255	3.78	-0.65	(-1.11,-0.18)	0.0065	nt1080	20.91	-0.31	(-0.52,-0.10)	0.0043
nt2678	6.05	-0.48	(-0.85,-0.10)	0.0135	nt285	37.78	-0.23	(-0.41,-0.05)	0.0117	nt1101	1.51	-1.06	(-1.73,-0.40)	0.0019
nt2684	6.55	-0.68	(-1.02,-0.33)	0.0001	nt287	2.02	-0.70	(-1.31,-0.09)	0.0246	nt1123	41.31	-0.22	(-0.40,-0.04)	0.0147
nt2690	9.57	-0.34	(-0.64,-0.04)	0.0268	nt294	9.07	-0.52	(-0.82,-0.22)	0.0007	nt1126	22.17	-0.52	(-0.73,-0.31)	<0.0001
nt2699	55.67	-0.23	(-0.40,-0.05)	0.0114	nt327	2.52	-0.73	(-1.31,-0.15)	0.0134	nt1138	9.07	-0.64	(-0.94,-0.33)	<0.0001

Nucleotide (nt) position	Freq. of variant SNP	β	95% CI	P
nt1139	4.79	-0.48	(-0.87,-0.08)	0.0184
nt1239	1.51	0.86	(0.16,1.55)	0.0154
nt1242	19.14	-0.38	(-0.61,-0.16)	0.0007
nt1243	1.76	-0.74	(-1.38,-0.10)	0.0242
nt1332	3.27	-0.70	(-1.19,-0.22)	0.0044
nt1341	3.53	-0.53	(-0.99,-0.07)	0.0234
nt1353	1.76	-1.24	(-1.84,-0.64)	0.0001
nt1359	3.78	-0.46	(-0.91,-0.01)	0.0443
nt1362	2.02	0.69	(0.10,1.27)	0.0215
nt1371	28.97	-0.22	(-0.41,-0.03)	0.0247
nt1479	3.02	-0.71	(-1.23,-0.19)	0.0075
nt1485	2.27	-0.55	(-1.10,-0.00)	0.0499
nt1509	3.78	-0.60	(-1.04,-0.16)	0.0072
nt1512	1.76	0.73	(0.07,1.38)	0.0296
nt1518	3.02	-0.58	(-1.08,-0.09)	0.0217
**HBeAg-negative, subgenotype Ce**				
nt2441	16.42	-0.74	(-1.44,-0.04)	0.0378
nt2607	11.94	-0.99	(-1.79,-0.20)	0.0155
nt2666	89.55	0.85	(0.03,1.68)	0.0425
nt2889	88.06	0.94	(0.15,1.73)	0.0206
nt53	32.84	0.77	(0.21,1.34)	0.0082
nt293	88.06	-1.10	(-1.93,-0.26)	0.0112
nt552	10.45	1.00	(0.11,1.90)	0.0291
nt592	22.39	0.75	(0.10,1.39)	0.0234
nt791	89.55	-1.25	(-2.24,-0.27)	0.0135
nt852	8.96	1.14	(0.22,2.05)	0.0159
nt921	10.45	0.93	(0.08,1.77)	0.0324
nt951	91.04	-1.43	(-2.30,-0.56)	0.0017
nt1126	11.94	-0.94	(-1.73,-0.15)	0.0198
nt1221	50.75	-0.68	(-1.21,-0.15)	0.0128
nt1229	43.28	-0.66	(-1.19,-0.12)	0.0167
nt1230	37.31	-0.59	(-1.14,-0.03)	0.0389
nt1383	10.45	1.17	(0.25,2.09)	0.0131
nt1574	31.34	-1.06	(-1.59,-0.52)	0.0002

CI, confidence interval; Freq, frequency; HBeAg, hepatitis B e antigen; HBV, hepatitis B virus.
