# Supplementary material for: Impact of Genetic Heterogeneity in Polymerase of Hepatitis B Virus on Dynamics of Viral Load and Hepatitis B Progression
Source: PLoS One. 2013 Jul 30;8(7):e70169. doi: 10.1371/journal.pone.0070169 (PMC3728348; doi:10.1371/journal.pone.0070169)
Supplement: Table S5 — Clusters of viral load-associated viral SNPs identified from principal component analysis, corresponding aa substitutions for these SNPs, and relationships of aa changes to sequence variation in overlapping or flanking T-cell epitopes, dynamics of viral load, and phase of chronic HBV infection. (PDF) [file pone.0070169.s008.pdf]

**Table S5. Clusters of viral load-associated viral SNPs identified from principal component (PC) analysis, corresponding amino acid (aa) substitutions for these SNPs, and relationships of aa changes to sequence variation in overlapping or flanking T-cell epitopes, dynamics of viral load, and phase of chronic HBV infection.**

| PC | Nucleotide (nt) position | Deduced aa positions (consensus aa)[a] | aa Substitution variants: Effect on viral load: β (change in log HBV DNA copies/mL)[b] | P-value | Frequency (%) by the phase of HBV-infection: IT | IC | LR | ENH | P-value for trend | Epitope aa location (bold)[c](HLA[d]) |
|---|---|---|---|---|---|---|---|---|---|---|
| Subgenotype Ba | | | | | | | | | | |
| PC1 | nt2699 | Pol 131 (P) | 0.07 | 0.8208 | 2.4 | 16.7 | 3.2 | 3.9 | 0.9680 | **Pol 137-145** (A*1101); **Pol 140-148** (A*2402); **Pol 145-159** |
| | nt2712 | Pol 136 (H) | 0.38 | 0.0005 | 63.4 | 66.7 | 42.2 | 38.5 | 0.0064 | (DRB1*0405, *0901, *1101, *1201, *1501, DRB4*0101, |
| | nt2771 | Pol 155 (G) | 1.03 | 0.3070 | 0 | 0 | 0.5 | 0 | 0.7606 | DRB5*0101); **Pol 147-156** (A*0201); **Pol 150-159** (A*1101) |
| PC2 | nt766 | Pol 559 (S) | -0.52 | 0.0104 | 2.4 | 0 | 8.4 | 7.7 | 0.1920 | **Pol 549-557** (A*0201, *0203, *0206); **Pol 562-570** (A*0201) |
| | nt771 | Pol 560 (V) | -0.37 | 0.6259 | 0 | 0 | 0.5 | 0 | 0.7606 | |
| | nt774 | Pol 561 (Q) | -0.92 | <0.0001 | 0 | 0 | 6.0 | 7.7 | 0.0840 | |
| | | S 204 (S) | -0.56 | <0.0001 | 2.4 | 0 | 29.5 | 19.2 | 0.0015 | **S 195-210** (DR1, DR7, DR13); **S 197-205** (A*0201); |
| | | S 206 (Y) | -1.41 | <0.0001 | 0 | 0 | 5.7 | 3.9 | 0.1706 | **S 199-208** (A*2402); **S 200-215** (DRB1*0301); **S 207-216** (A*0201) |
| | | S 207 (N) | -1.02 | <0.0001 | 0 | 0 | 6.8 | 7.7 | 0.0779 | |
| PC3 | nt110 | Pre-S2 41 (A) | -0.69 | 0.0006 | 2.4 | 0 | 8.7 | 7.7 | 0.1851 | **Pre-S2 27-46** (Class II); **Pre-S2 37-55** (Class II); **Pre-S2 44-53** (A2) |
| | nt114 | Pre-S2 42 (I) | -0.75 | 0.0004 | 0 | 33.3 | 6.5 | 11.5 | 0.1355 | |
| | nt123 | Pre-S2 45 (I) | -0.68 | 0.0006 | 2.4 | 0 | 8.9 | 11.5 | 0.1032 | |
| | nt126 | Pre-S2 46 (L) | -0.41 | 0.0070 | 2.4 | 0 | 16.5 | 19.2 | 0.0117 | |
| PC4 | nt1123 | Pol 678 (S) | -0.39 | 0.0005 | 12.2 | 33.3 | 43.2 | 46.2 | 0.0002 | **Pol 664-678** (DRB1*0405, *0901, *1101, *1501, DRB3*0101, |
| | nt1126 | Pol 679 (K) | -0.50 | <0.0001 | 9.8 | 33.3 | 36.5 | 42.3 | 0.0006 | DRB4*0101, DRB5*0101); **Pol 665-674** (A*1101) |
| PC5 | nt2583 | Pol 93 (K) | -0.36 | 0.0020 | 12.2 | 0 | 33.5 | 30.8 | 0.0067 | **Pol 96-110** (DRB1*0301, *0405, *1101, *1501, DRB4*0101, |
| | nt2618 | Pol 104 (N) | -0.40 | 0.0005 | 4.9 | 0 | 37.8 | 46.2 | <0.0001 | DRB5*0101) |
| PC7 | nt273 | Pol 394 (Q) | 1.03 | 0.3070 | 0 | 0 | 0.5 | 0 | 0.7606 | **Pol 385-399** (DRB1*0301, *0405, *1501, DRB3*0101, DRB4*0101); |
| | nt285 | Pol 398 (G) | NA | NA | NA | NA | NA | NA | NA | **Pol 388-397** (A*1101) |
| | | S 40 (N) | -0.44 | <0.0001 | 7.3 | 0 | 43.2 | 57.7 | <0.0001 | **S 31-50** (A*0201, DR1); **S 33-52** (Class II); **S 34-42** (A24); |
| | | S 44 (G) | -0.45 | <0.0001 | 7.3 | 66.7 | 38.9 | 23.1 | 0.0061 | **S 37-51** (DR1, DRB1*1101); **S 41-49** (A*0201) |
| PC10 | nt2684 | Pol 126 (D) | -0.44 | 0.0846 | 4.9 | 0 | 5.1 | 0 | 0.7156 | **Pol 115-123** (A*2402) |
| | nt2690 | Pol 128 (G) | 1.03 | 0.3070 | 0 | 0 | 0.5 | 0 | 0.7606 | |
| PC17 | nt1077 | Pol 662 (Q) | -1.00 | 0.0120 | 0 | 0 | 1.9 | 7.7 | 0.0918 | **Pol 651-661** (B*5101, *5401); **Pol 653-661** (A*0201, *0203, *0206); |
| | nt1080 | Pol 663 (A) | 0.99 | 0.0036 | 7.3 | 0 | 2.2 | 0 | 0.0364 | **Pol 664-678** (DRB1*0405, *0901, *1101, *1501, DRB3*0101, |
| | | | | | | | | | | DRB4*0101, DRB5*0101); **Pol 665-674** (A*1101) |

ENH, HBeAg negative hepatitis; HBV, hepatitis B virus; HLA, human leukocyte antigen; IT, immune-tolerant; IC, immune-clearance; LR, non/low-replicative; Pol, polymerase; S, surface; SNPs, single nucleotide polymorphisms.

Note: NA, Not applicable when only consensus aa is present despite of change in nucleotide sequence.

[a]Consensus in the present cohort.

[b]Results were derived from a linear mixed model adjusting for age.

[c]Defined at http://www.immuneepitope.org/.

[d]HLA alleles associated with the epitope. For specific HLA class I and DRB1 alleles, only those with a frequency >3% in Chinese according to the database of Tzu Chi Taiwan Marrow Donor Registry that contains HLA typing data from 46915 Taiwanese healthy volunteer are shown [1].

**Table S5. (Continued).**

| PC | Nucleotide (nt) position | Deduced aa positions (consensus aa)[a] | aa Substitution variants: Effect on viral load: β (change in log HBV DNA copies/ml)[b] | P-value | Frequency (%) by the phase of HBV-infection: IT | IC | LR | ENH | P-value for trend | Epitope aa location (bold)[c](HLA[d]) |
|---|---|---|---|---|---|---|---|---|---|---|
| Subgenotype Ce | | | | | | | | | | |
| PC1 | nt31 | Pre-S2 14 (D) | -1.77 | 0.0059 | 0 | 0 | 17.2 | 0 | 0.1414 | **Pre-S2 1-13** (Class II); **Pre-S2 1-14** (DR3, DR7, DR13); **Pre-S2 1-15** (DR2); **Pre-S2 1-26** (DR1); **Pre-S2 1-27** (Class II); **Pre-S2 10-25** (Class II); **Pre-S2 12-20** (A*0201); **Pre-S2 14-24** (Class II); **Pre-S2 16-33** (A*0201, DR1, DR2); **Pre-S2 17-34** (Class II) |
| | nt52 | Pre-S2 21 (Y) | -1.39 | 0.0258 | 0 | 0 | 17.2 | 11.1 | 0.0585 | |
| | nt915 | Pol 608 (Q) | NA | NA | NA | NA | NA | NA | NA | **Pol 616-630** (DRB1*0405, *0901, *1101, *1501, DRB4*0101, DRB5*0101) |
| | nt934 | Pol 615 (L) | -1.93 | <0.0001 | 4.8 | 33.3 | 65.5 | 55.6 | <0.0001 | |
| | nt1221 | Pol 710 (I) | -0.46 | 0.6248 | 4.8 | 16.7 | 5.2 | 0 | 0.6522 | **Pol 694-708** (DRB1*0301, *0405, *1501, DRB3*0101, DRB4*0101) |
| | nt1230 | Pol 713 (Q) | -0.00 | 0.9913 | 71.4 | 83.3 | 51.7 | 11.1 | 0.0048 | |
| PC2 | nt955 | Pol 622 (L) | NA | NA | NA | NA | NA | NA | NA | **Pol 616-630** (DRB1*0405, *0901, *1101, *1501, DRB4*0101, DRB5*0101); **Pol 623-631** (A*1101) |
| | nt993 | Pol 634 (Q) | NA | NA | NA | NA | NA | NA | NA | |
| PC3 | nt29 | Pre-S2 14 (D) | -1.77 | 0.0059 | 0 | 0 | 17.2 | 0 | 0.1414 | **Pre-S2 1-13** (Class II); **Pre-S2 1-14** (DR3, DR7, DR13); **Pre-S2 1-15** (DR2); **Pre-S2 1-26** (DR1); **Pre-S2 1-27** (Class II); **Pre-S2 10-25** (Class II); **Pre-S2 12-20** (A*0201); **Pre-S2 14-24** (Class II); **Pre-S2 16-33** (A*0201, DR1, DR2); **Pre-S2 17-34** (Class II) |
| | nt36 | Pre-S2 16 (R) | -1.43 | 0.0077 | 0 | 0 | 25.9 | 11.1 | 0.0263 | |
| | nt55 | Pre-S2 22 (F) | -0.39 | 0.4007 | 0 | 16.7 | 34.5 | 44.4 | 0.0009 | |

ENH, HBeAg negative hepatitis; HBV, hepatitis B virus; HLA, human leukocyte antigen; IT, immune-tolerant; IC, immune-clearance; LR, non/low-replicative; Pol, polymerase; S, surface; SNPs, single nucleotide polymorphisms.

Note: NA, Not applicable when only consensus aa is present despite of change in nucleotide sequence.

[a]Consensus in the present cohort.

[b]Results were derived from a linear mixed model adjusting for age.

[c]Defined at http://www.immuneepitope.org/.

[d]HLA alleles associated with the epitope. For specific HLA class I and DRB1 alleles, only those with a frequency >3% in Chinese according to the database of Tzu Chi Taiwan Marrow Donor Registry that contains HLA typing data from 46915 Taiwanese healthy volunteer are shown [1].

## Reference

1. Lai MJ, Wen SH, Lin YH, Shyr MH, Lin PY, et al. (2010) Distributions of human leukocyte antigen-A, -B, and -DRB1 alleles and haplotypes based on 46,915 Taiwanese donors. Hum Immunol 71: 777-82.
