# Supplementary material for: Impact of Genetic Heterogeneity in Polymerase of Hepatitis B Virus on Dynamics of Viral Load and Hepatitis B Progression
Source: PLoS One. 2013 Jul 30;8(7):e70169. doi: 10.1371/journal.pone.0070169 (PMC3728348; doi:10.1371/journal.pone.0070169)
Supplement: Table S6 — Shannon entropy values at each of the seven nucleotide positions in association with HCC development: comparison between participants with progression to HCC and non-progressors. (PDF) [file pone.0070169.s009.pdf]

Table S6. Shannon entropy values at each of the seven nucleotide positions in association with Hepatocellular Carcinoma (HCC) development: comparison between participants with progression to HCC and non-progressors.

Position	HCC	Non-HCC	Ratio HCC: Non-HCC
Subgenotype Ba	N=33	N=427	
Nt 3031	0.4045	0.0596	6.79
Nt 3150	0.6272	0.1595	3.93
Nt 3211	0.3693	0.1046	3.53
Nt 3213	0.3693	0.0463	7.98
Nt 27	0.5693	0.1201	4.74
Nt 1008	0.4045	0.1298	3.12
Subgenotype Ce	N=30	N=65	
Nt 552	0.6390	0	

HBV, hepatitis B virus; Nt, nucleotide.
