# Supplementary material for: Impact of Genetic Heterogeneity in Polymerase of Hepatitis B Virus on Dynamics of Viral Load and Hepatitis B Progression
Source: PLoS One. 2013 Jul 30;8(7):e70169. doi: 10.1371/journal.pone.0070169 (PMC3728348; doi:10.1371/journal.pone.0070169)
Supplement: Methods S1 — Direct sequencing, evaluation of nucleotide substitution models, and construction of consensus sequence. (PDF) [file pone.0070169.s010.pdf]

Methods S1

Direct sequencing

We performed nested PCR to amplify the polymerase gene from peripheral blood using the Advantage® 2 polymerase mix (Clontech Laboratories, Mountain View, CA) for high-fidelity amplification according to previously described method [1,2] with modification. The first-round PCR was performed to obtain a full-length amplicon using the primer pair NP1. When the PCR amplicon was not visible on a 1.5% agarose gel, amplification was performed by using two different primer pairs NP2 and NP3 to yield two overlapping fragments, 2126-bp and 1826-bp length, respectively. The first-round PCR product was further amplified in a nested PCR to yield two fragments, 1395-bp and 1366-bp length, respectively. The PCR products were directly sequenced with the BigDye® Terminator v3.1 Cycle Sequencing kit (Applied Biosystems, Foster City, CA). Primer design and amplification conditions are shown in Supporting Information Table S1.

Evaluation of nucleotide substitution models

After evaluating 24 major substitution models according to the goodness of fit of each model to the data measured by hierarchical likelihood ratio test and the Akaike information criterion, the best-fit model was the general time-reversible model allowing both a proportion of invariant sites and heterogeneity of rates across sites modeled by a γ distribution (GTR+I+G) (Supporting Information Table S2). Consideration of model selection uncertainty and multi-model inference should lead to equal or better phylogenies. Different models were used for phylogenetic analysis.

Consensus sequence

We determined the population consensus sequence for subgenotype Ba and Ce, separately, according to the most common presentation of the nucleotide consensus sequence, which is a one-string consensus sequence showing predominance of the nucleotides at every position. The consensus sequence of Ba is based on all 460 Ba sequences, and the consensus of Ce is based on all 95 Ce sequences. Consensus sequences were obtained using BioEdit v7.1.3.0. The resulted consensus sequence consists of nucleotides that occurred in more than 50% of subjects at almost all positions throughout the sequence region except 6 positions, which are nt1368 (frequency of predominant type: 42.8%), nt2699 (47.0%), nt2771 (48.3%), and nt3097 (49.8%) for the consensus of Ba; nt1229 (48.4%), nt1479 (37.9%), and nt2699 (44.2%) for the consensus of Ce. The consensus sequence for HBV/Ba is most closely related to reference sequences of strains (p-distance<0.005) isolated from China (AB073833), Taiwan (AB073840), Hong Kong (AB073828), and Vietnam (AF121246), while the consensus sequence for HBV/Ce is most closely related to reference sequences of strains (p-distance<0.012) isolated from China (AF458664), Japan (AB050018), and Korea (AY247031).

Reference

1. Sung JJ, Tsui SK, Tse CH, Ng EY, Leung KS, et al. (2008) Genotype-specific genomic markers associated with primary hepatomas, based on complete genomic sequencing of hepatitis B virus. J Virol 82: 3604-11.
2. Liu CJ, Chen PJ, Lai MY, Kao JH, Chen DS (2001) Hepatitis B virus variants in patients receiving lamivudine treatment with breakthrough hepatitis evaluated by serial viral loads and full-length viral sequences. Hepatology 34: 583-9.
