# Supplementary material for: Impact of Genetic Heterogeneity in Polymerase of Hepatitis B Virus on Dynamics of Viral Load and Hepatitis B Progression
Source: PLoS One. 2013 Jul 30;8(7):e70169. doi: 10.1371/journal.pone.0070169 (PMC3728348; doi:10.1371/journal.pone.0070169)
Supplement: Results S1 — Cumulative incidences of HCC by phases of natural history of chronic hepatitis B in the subcohort. (PDF) [file pone.0070169.s011.pdf]

Results S1

The 575 subjects whose sequence data were available and the entire sample were comparable with respect to all the baseline characteristics and follow-up clinical parameters across phase of natural history (Table 1 & Supporting Information Table S3).

Using the subcohort of 1054 subjects we estimated the 15-year cumulative incidences of hepatocellular carcinoma were 5.2% (95% confidence interval [CI]: 0.2%-10.2%) in IT, 26.3% (95% CI: 6.5%-46.1%) in IC, 4.7% (95% CI: 3.3%-6.1%) in LR, and 18.1% (8.4%-27.8%) in ENH.
